# Mutant p53 cancers reprogram macrophages to tumor supporting macrophages via exosomal miR-1246

**DOI:** 10.1038/s41467-018-03224-w

**Published:** 2018-02-22

**Authors:** Tomer Cooks, Ioannis S. Pateras, Lisa M. Jenkins, Keval M. Patel, Ana I. Robles, James Morris, Tim Forshew, Ettore Appella, Vassilis G. Gorgoulis, Curtis C. Harris

**Affiliations:** 1 Laboratory of Human Carcinogenesis, NCI-CCR, National Institutes of Health, Bethesda, 20892-4258 MD USA; 2 Molecular Carcinogenesis Group, Department of Histology and Embryology, School of Medicine, National Kapodistrian University of Athens, 75 Mikras Asias St, Athens, GR-11527 Greece; 3 Laboratory of Cell Biology, National Cancer Institute, National Institutes of Health, Bethesda, 20892-4258 MD USA; 4 Addenbrooke’s Hospital, Hills Road, Cambridge, CB2 0QQ UK; 5 Cancer Research UK, Cambridge Research Institute, Robinsons Way, Cambridge, CB2 0RE UK; 6 UCL Cancer Institute, Huntley St, Camden Town, London, WC1E 6DD UK; 7 Biomedical Research Foundation of the Academy of Athens, 4 Soranou Ephessiou St., GR-11527 Athens, Greece; 8 Faculty of Biology, Medicine and Health, University of Manchester, Manchester Academic Health, Science Centre, Wilmslow Road, Manchester, M20 4QL UK

## Abstract

TP53 mutants (mutp53) are involved in the pathogenesis of most human cancers. Specific mutp53 proteins gain oncogenic functions (GOFs) distinct from the tumor suppressor activity of the wild-type protein. Tumor-associated macrophages (TAMs), a hallmark of solid tumors, are typically correlated with poor prognosis. Here, we report a non-cell-autonomous mechanism, whereby human mutp53 cancer cells reprogram macrophages to a tumor supportive and anti-inflammatory state. The colon cancer cells harboring GOF mutp53 selectively shed miR-1246-enriched exosomes. Uptake of these exosomes by neighboring macrophages triggers their miR-1246-dependent reprogramming into a cancer-promoting state. Mutp53-reprogammed TAMs favor anti-inflammatory immunosuppression with increased activity of TGF-β. These findings, associated with poor survival in colon cancer patients, strongly support a microenvironmental GOF role for mutp53 in actively engaging the immune system to promote cancer progression and metastasis.

## Abstract (web summary)

p53 gain of function mutants (mutp53) are involved in the pathogenesis of most human cancers. Here, the authors show that mutp53 regulates the tumor microenvironment by inducing the release of specific exosomes containing miR-1246 that once received by macrophages turns them into tumor supportive macrophages.

## Introduction

Exosomes are small spherical packages and one of the vesicle types released by cells into the extracellular environment. Exosomes convey information to neighboring or remote cells by delivering RNAs and proteins thus affecting signaling pathways in various physiological and pathological conditions including cancer^1,2^. The production of exosomes and the molecular cargo they carry are affected by external signals such as oxidative stress and ionizing radiation^3,4^. Therefore, p53, a cellular stress responsive transcription factor, plays a major role in exosome machinery and release while under microenvironmental stress. For instance, p53-dependent regulation of TSAP6 was reported to govern exosome secretion and content^5,6^.

Mutations in the *TP53* gene (encoding for the p53 protein) are one of the most frequent genetic alterations in human cancer^7–9^. Besides the abrogation of the wild-type (WT) p53-mediated tumor suppression, a distinct set of missense mutations was reported to endow mutant p53 (mutp53) proteins with novel activities termed gain-of-function (GOF). Such GOF activities dramatically alter tumor cell characteristics, primarily through their interactions with other cellular proteins and regulation of cancer cell transcriptional programs^10–13^. On a cellular level, increased mutp53 protein stability leads to a substantial intracellular mutp53 accumulation in cancer cells, further disrupting cellular homeostasis and creating oncogenic stress^14,15^. Thus, cancer cells appear to be addicted to high levels of mutp53 for their survival and oncogenic properties. In this study, we hypothesized that in addition to its cell-autonomous GOF mechanisms, mutp53 might affect microenvironmental conditions by facilitating the release of exosomes stemming from mutp53-dependent cellular stress.

In most solid cancers, a major component of the tumor stroma are macrophages referred to as tumor-associated macrophages (TAMs)^16^ that are mostly derived from peripheral blood monocytes recruited into the tumor mass^17–20^. In recent years, TAMs have been extensively studied and proposed as a significant contributing factor to tumor progression. The communication between tumor cells and macrophages was suggested to be mediated via exosomal transfer where packaged proteins and microRNAs (miRs) were reported to immunomodulate the macrophages at the receiving end^21–23^.

In this study, we discovered a microenvironmental GOF mechanism for mutant p53 by driving exosome-based communication between tumor and immune cells forming a distinct sub-population of tumor supportive macrophages. Our findings identify miR-1246 as a unique cargo of mutp53-derived exosomes potentially amenable for therapeutic and diagnostic applications in colon cancer.

## Results

### Tumor cells harboring mutp53 reprogram macrophages

**Fig. 1:**
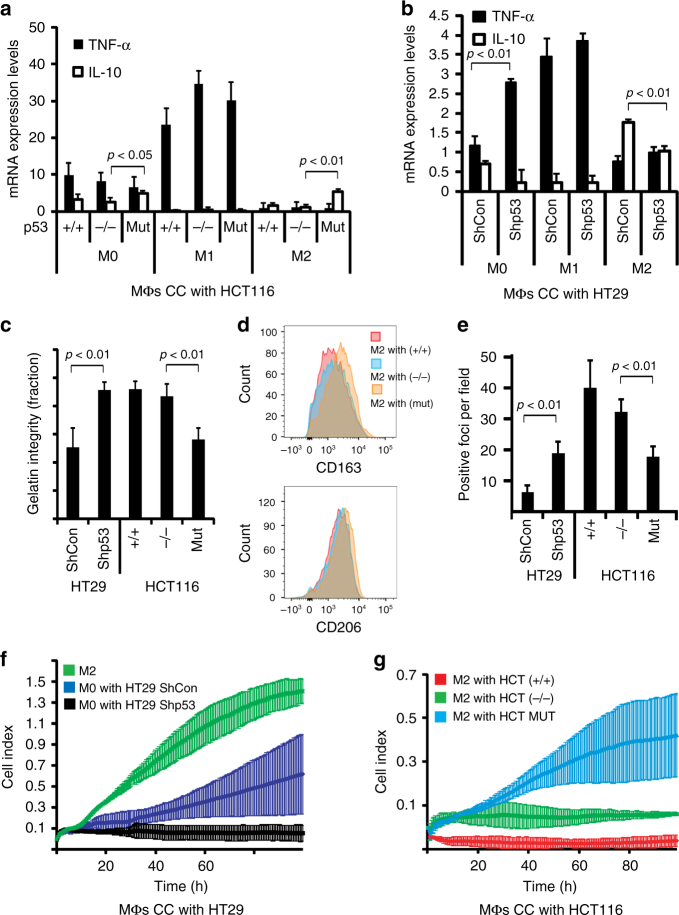
Carcinoma cells harboring mutp53 exert a non-cell-autonomous effect over macrophages. **a** Primary human monocytes were grown and differentiated towards three different lineages of macrophages (M0, M1, and M2) and co-cultured with an isogenic set of HCT116 cells differing by their p53 status (+/+ = WT p53, −/− = p53 null, mut = mutp53, p.R248W). RNA was extracted and subjected to qPCR analysis with primers specific to TNF-α and IL-10. Values were normalized for GAPDH mRNA in the same sample. **b** Primary monocytes were grown as in **a** and co-cultured with HT29 (mutp53- R273H) cells that underwent stable shRNA knock-down using scrambled oligo (ShCon) or specific to p53 (Shp53). TNF-α and IL-10 were analyzed as in **a**. **c** Co-cultured macrophages were seeded onto a cy-3-gelatin covered glass slide for 48 h and gelatin degradation rates were measured. **d** Co-cultured macrophages were harvested, stained with fluorescent antibodies against CD163 and CD206 and analyzed by flow cytometry. Relative intensities were compared with isotype controls. **e** Co-cultured macrophages were seeded in an eight-well chamber slide and incubated with fluorescent zymosan particles for 24 h, after which zymosan phagocytosis was evaluated. **f**,**g** Co-cultured macrophages were harvested and reseeded in an electrical-impedance monitoring chamber for 5 days to measure either migration (**f**) or invasion (**g**) properties. All experiments in this figure were repeated three times, error bars represent standard errors

We investigated the mechanism by which tumor cells harboring specific missense mutations in the *TP53* gene (mutp53) might reprogram neighboring macrophages. In the initial human cell co-culture experiment, both cultures were separated by a membrane allowing the transport of molecules and particles less than 0.4 µm in size. The macrophage culture originated from CD14^+^ primary human monocytes (Supplementary Fig. 1a, b), which were activated by three different stimulatory cytokine cocktails to derive either M0 macrophages (not polarized), M1 macrophages (classically activated), or M2 macrophages (alternatively activated). Polarization patterns were validated by conducting a gene expression array for M1 and M2 polarized primary macrophages (Supplementary Table 1). For the carcinoma cell compartment of the co-culture, we selected several cellular models where mutp53 was either expressed (the R248W mutant in HCT116 cells), induced (the V157F, R175H, R273H or R249S in H358 cells), or knocked-down (the R273H in HT29 cells) (Supplementary Fig. 1c). We monitored the effect of mutp53 on the co-cultured macrophages using a set of cytokines previously reported to be altered in the TAM equilibrium^24^. After being exposed to tumor cells that harbor mutp53, M0 and M2 macrophages showed increased IL-10, CCL2, and VEGF while less TNF-α expression when compared with equivalent macrophages co-cultured with cells lacking mutp53 or in cells where mutp53 was knocked-down (Fig. 1a, b, Supplementary Fig. 1d). These results were further corroborated using ELISA assays showing secreted protein levels of IL-10, TNF-α, and CCL2 in reprogrammed macrophages (Supplementary Fig. 2a). Additional proteome profiling of secreted cytokines corroborated that when mutp53 is present in the tumor cells, M2 macrophages produce less pro-inflammatory cytokines such as IL-8, IFN-γ ICAM-1 (Supplementary Fig. 1e). Furthermore, the occupancy of both CD206 and CD163, two TAM markers, was found to be increased on the surface of mutp53-reprogrammed macrophages (Fig. 1d, Supplementary Fig. 1h). This TAM-like population was also characterized by an attenuated phagocytic capacity when incubated with microbial particles (Fig. 1e, Supplementary Fig. 1f). These reprogrammed macrophages also presented enhanced degradation of the extra cellular matrix (ECM) and became more motile and invasive when compared with macrophages that were introduced to tumor cells that did not carry any p53 mutation (Fig. 1c, f, g, Supplementary Fig. 1g). When macrophages were co-cultured with an inducible set of TP53 mutants in H358 cells, the p53 R273H or R249S mutant forms favored a similar phenotypic shift in the co-cultured macrophages, whereas other variants, such as p53 V157F or R175H, did not (Supplementary Fig. 1i), consistent with these phenomena being mutant-specific. Additionally, we used mass spectrometry to compare the secretome of M2 macrophages co-cultured with mutp53 or WT p53 tumor cells, or without any co-culture. When macrophages were exposed to tumor cells carrying mutp53, increased secretion of several pro-tumorigenic factors, such as matrix metallopeptidase 9 (MMP-9), vascular non-inflammatory molecule 1 (VNN-1), and transforming growth factor beta-induced (TGFBI), was observed (Supplementary Table 2). When compared with the M0 and M2 macrophages, very few of these paracrine effects (described in Fig. 1a–i) were observed in classically activated M1 macrophages, as their IL-10 and TNF-α balance was unaffected and their ability to perform phagocytosis and ECM degradation was unchanged.

### Exosomes shed from mutp53 tumor cells carry a specific miR signature

**Fig. 2:**
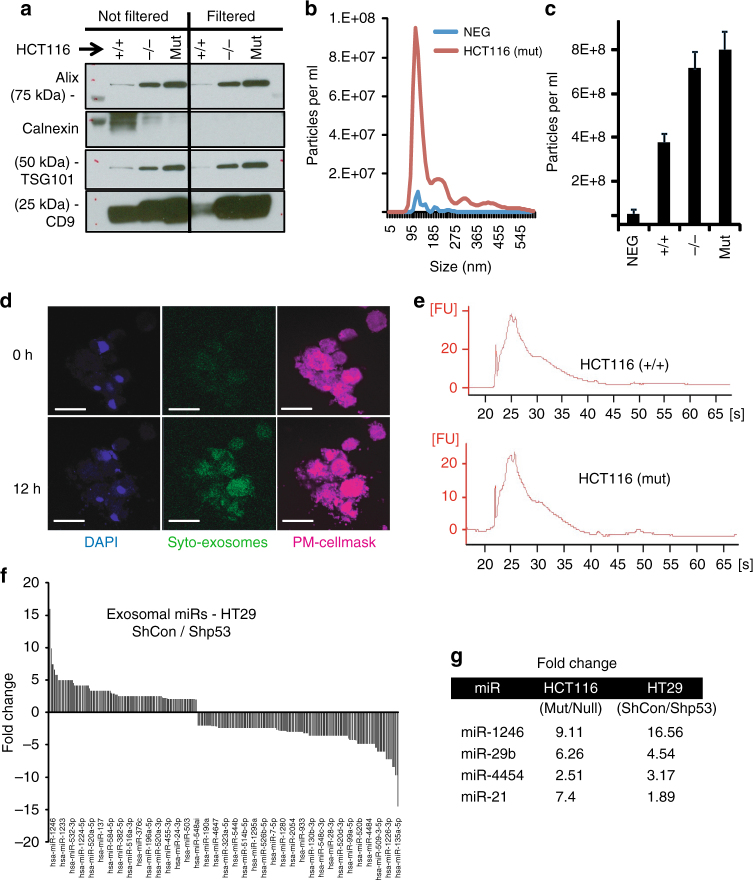
Exosomes shed from tumor cells are taken up by neighboring macrophages. **a** Exosomes were isolated from HCT116 cells harboring either WT, mutant (R248W), or no p53 as described in the Methods section. Isolations were either filtered (0.22 µm) or kept unfiltered during the procedure. Subsequently, isolations were lysed and subjected to western blot analysis with the indicated antibodies for exosomal markers. Calnexin served as a cellular contaminants marker. **b**,**c** Exosomes isolated from HCT116 cells underwent a nanoparticle tracking analysis (NTA) to determine exosomal size distribution (**b**) and concentration (**c**). The exosome samples were compared with cell-free medium that underwent similar isolation procedure (NEG). **d** Exosomes isolated from HCT116 cells were labeled using a Syto RNAselect dye before being incubated with macrophages for periods of 24 or 48 h. Accumulation of exosome uptake was captured in time-lapse movies. Macrophage nuclei are labeled with DAPI, plasma membranes are labeled with CellMask far-red. Bars = 25 µm. **e** RNA was extracted from HCT116-derived exosomes and its integrity and quality were tested using a bioanalyzer system before it was subjected to miRNA expression assay and normalized to the most abundant 100 miRs. **f** A representative comparison displaying greater than twofold changes in miRs between HT29 cells either knocked down for mutp53 (Shp53) or not (ShCon). **g** Changes in expression for four prominent miRs that were found to be significantly more abundant in mutp53 HCT116 and HT29 cells

Given that the mutp53-driven reprogramming of M0 and M2 macrophages shown in Fig. 1 occurred with no cell-to-cell contact, we hypothesized that specific sub-cellular materials are being exchanged between the tumor cells and the macrophages (including cytokines and extracellular vesicles). To explore the mechanism by which such intercellular communication is mediated, we focused on exosomes released by tumor cells harboring mutp53. Exosomes, cell-derived 30–150 nm vesicles, were reported to be involved in a plethora of intercellular interactions, including cancer promoting immune regulation and the formation of metastatic niches^1,5,25,26^. We therefore isolated and characterized exosomes from HCT116 and HT29 cells (Fig. 2a–c, Supplementary Fig. 2a−c). Western blot and mass spectrometry analyses confirmed the presence of exosomal marker proteins, including Alix, TSG-101, and CD9^22,27^. Purified exosomes were further filtered (0.22 µm) and used in all experiments. Calnexin was used as a marker demonstrating that there was no detectable cellular contamination in filtered isolations (Fig. 2a, Supplementary Fig. 2c). Nanoparticle tracking analysis (NTA) indicated a typical average exosome size distribution ranging from 102 to 112 nm; transmission electron microscopy (TEM) verified exosome morphology and size (Fig. 2b, c, Supplementary Fig. 2a, b). To demonstrate the exosomal transfer between tumor cells and macrophages, we labeled tumor-derived exosomes with Syto-RNAselect dye and incubated them with mature macrophages (Fig. 2d). Purified exosomes were subjected to RNA extraction, which yielded mostly small RNA species as revealed by the bioanalyzer measurement (Fig. 2e). Since microRNAs (miRs) were described as a major RNA species shuttled through exosomal transfer and activating signaling pathways at the receiving end^28^, we focused on their composition in exosomes released from carcinoma cells. An miR-chip array measuring the exosomal cargo of HCT116 and HT29 cells was performed, allowing the comparison between miRs that are abundant in exosomes shed by mutp53 tumor cells, WT p53 cells and null p53 cells (Fig. 2f, g). The results, which were normalized to the most highly expressed 100 miRs, uncovered a specific signature of miRs that were more abundant in exosomes from mutp53 cells as well as a different group of miRs that were over-expressed with the absence of a p53 mutation (Fig. 2g). Several prominent miRs annotated as mutp53-associated-miRs, such as miR-1246, miR-21, and miR-29b, were previously reported to be packaged into exosomes and transported between tumor cells and other cell types^2,21,29^. A comprehensive list of miRs is presented in Supplementary Table 5. To determine if the increased levels of miR-1246 and miR-21 in exosomes stem from increased levels of these miRs in the parent cells, we also compared the abundance of these miRs between HCT116 cells differing by their p53 status. Although these miRs were enriched in mutp53-cell-derived exosomes, the levels of miR-1246 and miR-21 were independent of the p53 status of the tumor cells as their levels were similar in cells harboring mutp53 and cells harboring WT p53 (Supplementary Fig. 2e). To verify that the identified miRs are associated directly with exosomes, we separated the mutp53-derived exosomal isolations into fractions using a sucrose density gradient technique. Both miR-21 and miR-1246 were observed to be expressed primarily in the Alix- and CD9-positive fractions, indicating a close association with exosomes for these miRs (Supplementary Fig. 2f, g). We also pre-treated the exosomes with RNAse to deplete any external RNA pulled down with the exosomes. Levels of miR-1246 were abolished completely in fraction 8, which was not associated with exosomal markers; however, in fractions 5–7, considerable levels of miR-1246 were detected even after exosomes were treated with RNAse indicating its presence inside the vesicles (Supplementary Fig. 2h).

### Exosomal miR-1246 is associated with mutp53 in tumor cells and TAMs

**Fig. 3:**
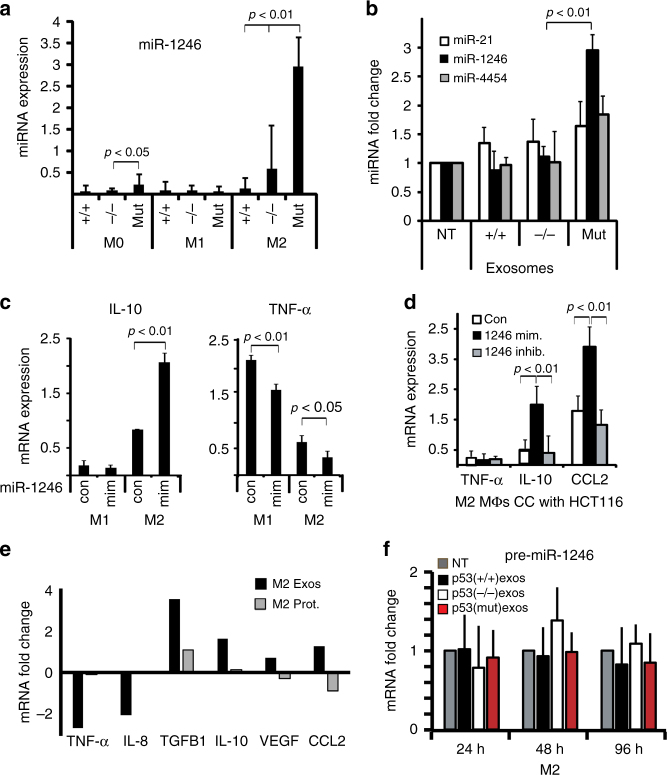
Exosomes shed from mutp53 tumor cells carry specific microRNA cargo. **a** Macrophages were co-cultured with HCT116 cells. RNA was extracted from the macrophages and subjected to qPCR analysis with primers specific to miR-1246. Values were normalized for RNU48 in the same sample. **b** Macrophages were grown in the presence of 10 µg exosomes isolated from HCT116 cells differing by their p53 status. miR-1246, miR-21, and miR-4454 levels were measured as in **a**. **c** M1 and M2 macrophages were transfected with LNA-miR-1246 mimic (mim) and compared with an equivalent control vector (con). RNA was extracted from the macrophages and subjected to qPCR analysis with primers specific to TNF-α and IL-10. **d** HCT116 mutp53 (R248W) cells were transfected with LNA-miR-1246 mimic (mim) or miR-1246 inhibitor (inhib.) and compared with an equivalent control vector (con). Forty-eight hours later, cells were co-cultured with M2 macrophages for an additional 3 days after which RNA was extracted from the macrophages and subjected to qPCR analysis with primers specific to TNF-α, CCL2, and IL-10. **e** Exosomes were isolated from HCT116 cells harboring mutp53 and separated from free proteins by a size-exclusion column. Macrophages were incubated with either pbs negative control (Con), the exosomes fraction (Exos) or the free proteins fraction (Prot.). RNA was extracted from the macrophages and subjected to qPCR analysis with primers specific to the indicated genes. Data are presented as fold changes compared with a PBS-treated control. **f** M2 macrophages were grown in the presence of 10 µg exosomes isolated from HCT116 cells differing by their p53 status for different time periods. RNA was extracted from the macrophages and subjected to qPCR analysis with primers specific to pre-miR-1246. All experiments in this figure were repeated three times, error bars represent standard errors

Next, we determined whether miR-1246 containing exosomes are transferred from tumor cells to neighboring macrophages. miR-1246 was found to be increased in the co-cultured M0 and M2 macrophages specifically after they had been exposed to tumor cells harboring mutp53 whereas tumor cells not carrying mutp53 did not exert such an effect (Fig. 3a). In addition, intracellular miR-1246 levels were increased only in the macrophages that were cultured with purified exosomes collected from mutp53 cells and not when cultured with exosomes from either WT p53 cells or p53 null cells (Fig. 3b). We validated these results by overexpressing miR-1246 in M2 macrophages using a locked-nucleic–acid (LNA)-based miR-1246 mimetic (miR-1246 mimic) that yielded macrophage reprogramming consistent with our co-culture results, including increased levels of IL-10 expression while TNF-α levels decreased (Fig. 3c, Supplementary Fig. 3a). Furthermore, inhibition of miR-1246 in co-cultured mutp53 tumor cells significantly decreased miR-1246 levels in derived exosomes (Supplementary Fig. 3b, *p* < 0.01 Student’s *t*-test) and attenuated the increase in IL-10 and CCL2 compared with HCT116 cells and HT29 treated with miR-1246 mimic (Fig. 3d, Supplementary Fig. 3d). Therefore, the indirect manipulation of exosomal miR-1246 sources had a paracrine effect over the macrophage polarization patterns consistent with the co-culture results. To ensure that macrophage reprogramming is driven by exosomes and not free protein/RNA released by mutp53 tumor cells, we used size-exclusion methods. As shown in Fig. 3e, exosomes are the major factor leading to a decrease in TNF-α and IL-8 levels as opposed to an upregulation of IL-10, TGFBI, VEGF, and CCL2. Exposure of the macrophages to free proteins pulled down with the exosomes did not promote such an effect. miR-1246 was found to be upregulated in M2 macrophages when treated with the exosomes fraction but not the proteins fraction (Supplementary Fig. 3c). In addition, we examined the possibility that endogenous miR-1246 levels are elevated in mutp53-reprogrammed macrophages, driving their phenotypic shift. When pre-miR levels of miR-1246 were measured, no significant difference was observed between non-treated macrophages (NT) and macrophages added with exosomes from HCT116 cells (p53(+/+), p53 (−/−) and p53(mut) exos) (Fig. 3f). On the other hand, as presented both in Fig. 3b and in Supplementary Fig. 3e, the mature miR-1246 was significantly increased 48 and 96 h post exosomes addition (*p* < 0.01, Student’s *t* test). These findings are consistent with an exogenous miR-1246 increase that drives macrophage reprogramming. Next, we aimed to determine the number of miR-1246 molecules transferred between tumor cells and macrophages via exosomes. To quantitate the amounts of miR-1246 copies per vesicle, we isolated 10 µg (protein) of HCT116 mutp53 exosomes and measured both particles concentration (using NTA) and RNA concentration (using Agilent Bio-analyser chips). Based on total RNA and small RNA levels it is clear that the majority of RNA in those exosomes is of small RNA species and almost 60% of these small RNAs are miRs (Supplementary Fig. 3f). We then subjected the exosomal RNA to absolute quantitation RT-PCR. Known quantities of the miR-1246 mimic were used to create a standard curve. Specifically, mir-1246 mimic was diluted to a concentration range of 1.386×10^12^–1.386×10^6^ copies per reaction and each point plotted is an average of triplicate fluorescence values for each standard concentration measured (Supplementary Fig. 3g, HCT116-derived exosomes are marked with an arrow). A synthetic non-human cel-miR-39 was spiked in during RNA isolation to assure equal efficiency of RNA processing between reactions. The converted number of miR-1246 molecules was divided by the number of exosomes measured using NTA. We evaluated an average of 8.8 ± 1.92 copies per exosome. These results are consistent with several recent publications suggesting that exosomes enriched with miRs and most specifically miR-1246 will carry a number varying from several to a few dozens of copies per vesicle^30,31^.

### Mutp53 triggers miR-1246-dependent reprogramming of macrophages

**Fig. 4:**
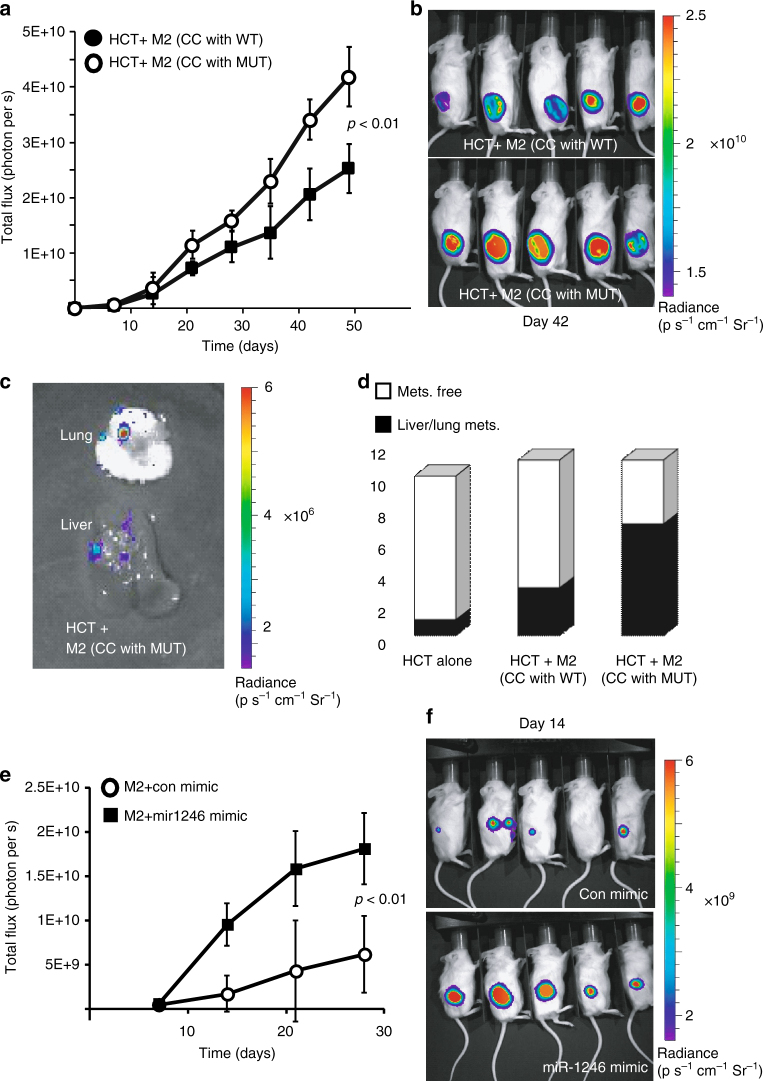
miR-1246 is associated with mutp53 and plays a role in reprogramming TAMs. **a**,**b** M2 macrophages were co-cultured with either mutp53 or WT p53 HCT116 cells for 6 days. Subsequently, 10^5^ reprogrammed macrophages were mixed with 5 × 10^5^ fresh HCT116 WT cells (carrying a luciferase vector) and co-injected subcutaneously into NOD-SCID mice. Each group consisted of 10 or 11 animals. Tumor development was monitored weekly. **c**,**d** On day 56 of the experiment described in **a**, **b**, mice were killed and their liver and lungs were monitored for metastatic foci (**c**), and the number of organs observed with metastases were compared with a group of mice injected with HCT116 cells alone (**d**). **e**,**f** M2 macrophages were transfected with LNA-miR-1246 mimic and compared with an equivalent control vector. Three days later, the transfected macrophages were mixed with luciferase expressing HCT116 cells and co-injected subcutaneously to the back of NOD-SCID mice (HCT116 + M2 with control mimic, *n* = 5, HCT116 + M2 with miR1246 mimic, *n* = 5). Mice were monitored weekly for tumor growth using an IVIS imager (**e**) and luminescent fluxes were quantified (**f**). Error bars represent standard errors

To evaluate the tumor supportive capacity of mutp53-reprogrammed macrophages, we co-cultured M2 macrophages with mutp53 HCT116 cells as described above. We then mixed the co-cultured macrophages with fresh tumor cells and co-injected them subcutaneously as xenograft tumors into NOD-SCID mice. Significantly, the co-injected mutp53-reprogrammed macrophages promoted the development of larger tumors (Fig. 4a, b) and increased metastatic burden (Fig. 4c, d, Supplementary Fig. 4b) compared with macrophages exposed to WT p53 tumor cells before being co-injected (*p* < 0.01, repeated measures ANOVA). When M1 macrophages were used in a similar experiment, no significant difference was recorded between the mutp53 reprogrammed group and the WT p53 reprogrammed group (Supplementary Fig. 5b). We determined cytokeratin20 levels in the xenografts to confirm HCT116 carcinoma presence (Supplementary Fig. 4d).^32^ In addition, we compared between different macrophages states (M0, M1, and M2) and concluded that M2 macrophages have a higher tumor supportive capacity when mixed with tumor cells compared with the M0 and M1 macrophages (Supplementary Fig. 4a). Additionally, we evaluated the tumor supportive effect of miR-1246 on TAMs. M2 macrophages were transfected with miR-1246 mimic and co-injected with HCT116 cells or HT29 cells to form xenograft tumors in NOD-SCID mice. Compared with the mimic control, tumors co-injected with M2 macrophages transfected with miR-1246 gave rise to significantly larger tumors (Fig. 4e, f, Supplementary Fig. 4e) and increased metastatic burden to the lung and liver (Supplementary Fig. 4d, e) (*p* < 0.01, repeated measures ANOVA). In line with our in vitro findings, when M2 macrophages were transfected with miR-1246 inhibitor, no significant difference was observed in tumor growth (Supplementary Fig. 4e). We also tested an orthotopic model focusing on the metastatic spread. Intra-splenic injections of HCT116 cells together with miR-1246-transfected M2 macrophages were used to directly assimilate metastatic foci in mice livers through the splenic vein. When compared with the control group injected with HCT116 and macrophages transfected with a control mimic, the miR-1246 transfected group was found with a significantly increased metastatic burden (Supplementary Fig. 4f–g) (*p* < 0.01, Student’s *t* test).

### Mutp53 positively correlates with TAMs in colorectal cancer patients

**Fig. 5:**
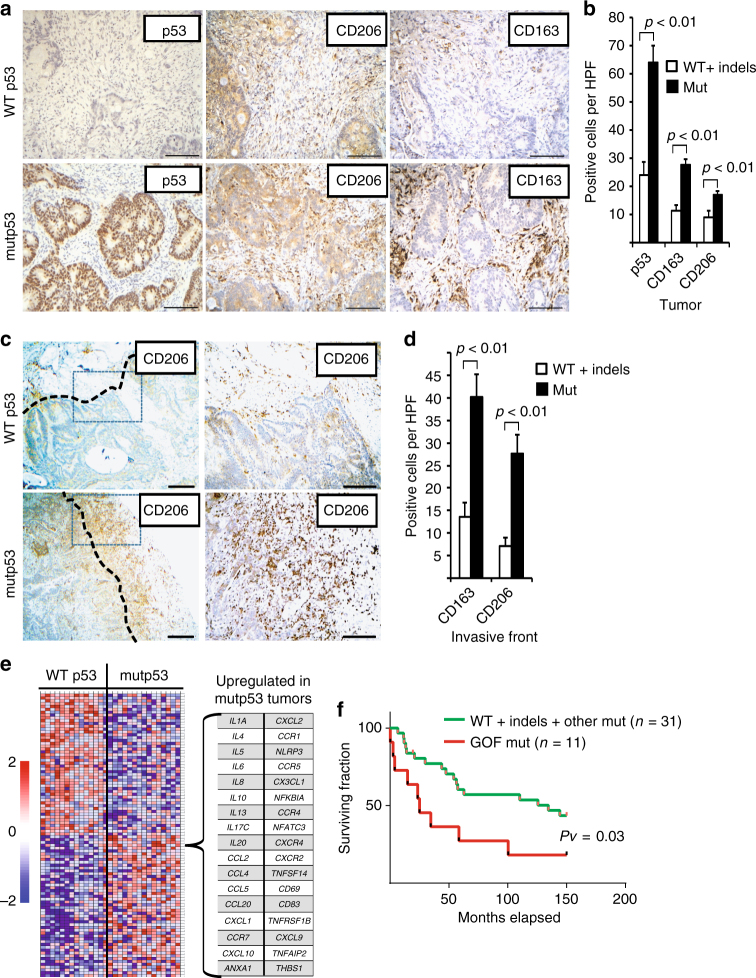
Mutp53 positively correlates with TAMs in CRC patients. **a** Strong epithelial immunostaining of p53 in the cancerous glands is correlated with diffuse and strong immunostaining of CD163 and CD206 in the surrounding stroma, in a representative human CRC case carrying mutp53 compared with a WT p53 case. Bars represent 100 µm. **c** Diffuse and strong CD206 immunopositivity in the invasive front of a mutp53 human CRC case as compared with a WT p53 case. The left panels display tumors at low magnification where the invasive front is marked with a dashed line. The boxed area is displayed in the right panels at a higher magnification. Bars represent 200 µm in the left panels and 100 µm in the right panels. **b**,**d** Staining abundance and intensity were calculated for all 42 specimens as described in the Methods section. Cases were grouped by p53 typing outcome—missense p53 mutations were defined as “mutp53 group”, while insertions, deletions and no mutations were defined as “WT+ indels group”. The analysis was conducted either for the entire cancerous tissue (tumor, **b**) or focused in tumor boundaries (invasive front, **d**). **e** RNA from frozen tissue samples of 29 CRC cases was extracted and subjected to mRNA expression microarray analysis. The heatmap depicts the expression patterns of the genes whose abundance was significantly upregulated or downregulated between the “WT + indels” (*n* = 13) group and the “mutp53” group (*n* = 16). A specific molecular signature of genes upregulated in mutp53 CRC cases is detailed. **f** Survival curve of CRC patients divided by TP53 status comparing between the WT+ indels combined with non-GOF p53 mutants (WT + indels + other mut) with patients carrying tumors with GOF mutations (GOF mut). Error bars represent standard errors

To evaluate the possible clinical relevance of our findings, we investigated surgical specimens from 43 colorectal cancer patients. Tagged Amplicon Deep Sequencing to an average depth of ×15,000 was used to sequence all exons of *TP53* and hence divide the tumors into those with a missense mutation in p53 (“mutp53” group, *n* = 29) or tumors with no missense p53 mutation (either WT or indels = “WT+ indels” group, *n* = 14) (Supplementary Fig. 5a). Immunohistochemistry (IHC) analysis showed an expected intense diffuse nuclear p53 staining that was more prominent in the mutp53 cases (Fig. 5a). Consistent with our co-culture findings, both TAM markers, CD163- and CD206-expressing cells, were found to be positively stained in the tumor stroma, particularly in the mutp53 cases when compared with tumors without missense p53 mutations (Fig. 5a, b, Supplementary Fig. 5c). Additionally, the CD163- and CD206-positive cells were prominent in the invasive front region of those tumors (Fig. 5c, d). Analysis of mRNA expression identified a signature of coding and non-coding genes upregulated in the mutp53 group of tumors using a GSEA platform (Fig. 5e). Among a general inflammatory molecular signature that was found to be overexpressed in the mutp53 group, a specific set of “alternative macrophage polarization” hallmark genes such as IL-4, IL-13, CCL2, and IL-10^18,33^, were upregulated, consistent with our in vitro observations.

When comparing the survival rates of patients with mutp53 tumors to the WT +  indels group of patients, no statistically significant difference was observed (Supplementary Fig. 5d). However, when we focused on a set of mutants that are classified as mutants with GOF activity^7,11,34^ (i.e. positions R245, R248, R175, R273, R282), we found a significantly positive correlation with poor survival even when grouping the WT + indels patients with other non-GOF mutants (Fig. 5f) (*p* = 0.03, Kaplan−Meier method; log-rank test). Furthermore, these GOF mutp53 specimens were observed to have a significant increased infiltration of CD206-positive macrophages at the invasive front of the tumors (Supplementary Fig. 5e) (*p* < 0.01, Student’s *t* test), as well as an overexpression of several inflammatory signatures (such as the IL-10 and TGF-β pathways) or oncogenic signatures (such as epithelial to mesenchymal transition and ECM remodeling) (Supplementary Table 3). Altogether, such observations are consistent with TP53 mutant-specific GOF^11,35,36^. While the lesions in this cohort might carry additional mutations, which can impact the tumor microenvironment and clinical outcome, there is a clear positive correlation between mutp53, TAMs, and survival. Therefore, an interplay between tumor cells harboring GOF p53 mutants with their microenvironment may result with a clonal selective pressure leading to poor prognosis.

### Mutp53 positively correlates with miR-1246 in CRC patients

**Fig. 6:**
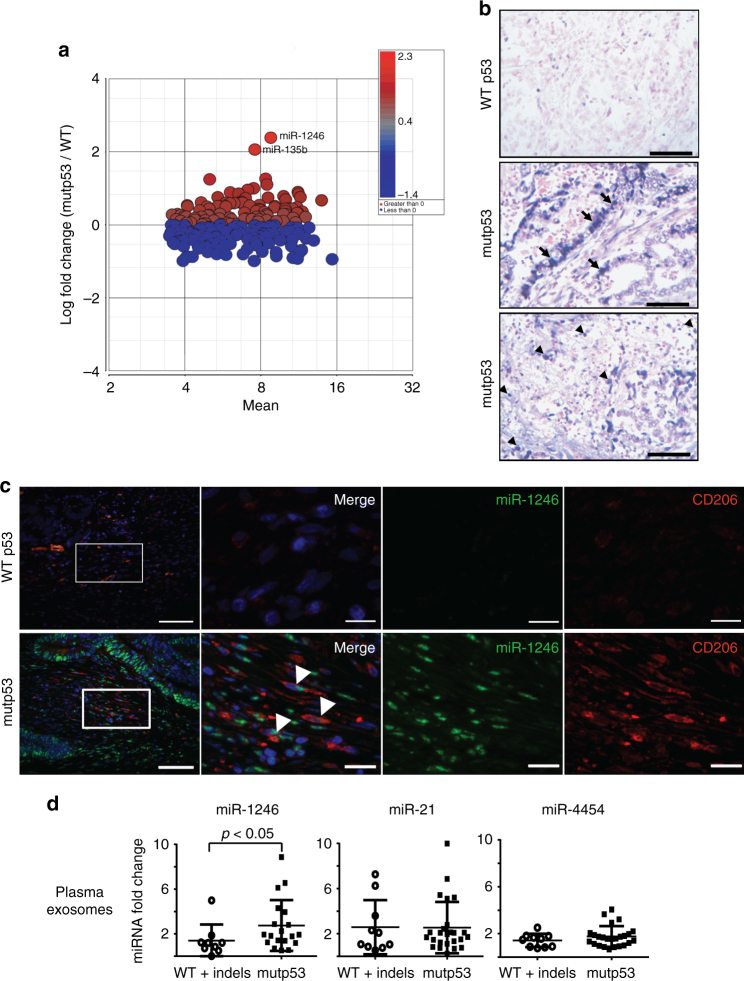
Mutp53 correlates with miR-1246 in CRC patients. **a** MicroRNA microarrays were used to profile microRNA expression levels in 27 WT p53 cases and 28 mutp53 cases. Complete table with *p *values is presented in the Supplementary Information (Supplementary Table 4). **b** Strong epithelial (middle panel) and stromal (lower panel) miR-1246 hybridization signal in mutp53 tumor compared with WT p53 tumor. Bars = 100 µm. **c** Co-detection of miR-1246 and CD206 in the stroma of sporadic colorectal carcinomas harboring mutp53 and WT p53. White arrowheads demonstrate miR-1246+/CD206+ cells. Scale bars: 200 μm (low magnification); 25 μm (high magnification). DAPI was used for nuclear stain (blue). **d** Exosomes were isolated from plasma of CRC patients categorized as “WT + indels” (*n* = 11) or “mutp53” (*n* = 25). RNA was extracted from the exosomes and miR-1246, miR-21, and miR-4454 levels were measured using qPCR. Quantification and normalization were performed using NTA and miR-454. Error bars represent standard errors

Next, we validated the association of miR-1246 with mutp53 in cancer patients. We performed a full microRNA profiling of RNA extracted from 27 WT p53 colorectal tumors and 28 mutp53 colorectal tumors. The analysis produced 219 miRs expressed in all tumors. When comparing the expression levels between WT and mutp53 tumors, miR-1246 was found to be the top miR associated with mutp53 tumors with a logarithmic fold change of 2.29 and *p*value of 0.045. (Fig. 6a, fully presented in Supplementary Table 4). To further characterize the correlation between mutp53 and miR-1246, we conducted in situ hybridization (ISH)^28^ of miR-1246 in the tumor tissues. Tumors harboring missense mutp53 presented significantly higher positive staining compared with WT p53 tumors (Fig. 6b, Supplementary Fig. 6a, b) (*p* < 0.01, Student’s *t* test). Importantly, miR-1246 was found both in the cancer cells compartment of the mutp53 tumors and in the stromal compartments including immune cells of monocytic appearance (Fig. 6b, Supplementary Fig. 6b: Arrows—cancer cells, arrowheads—non-epithelial cells). The ISH of miR-1246 was validated with scrambled negative control as well as with U6 snRNA serving as positive control (Supplementary Fig. 6b, lower panel). To validate that miR-1246 is transferred to macrophages in mutp53 colon cancers, we conducted a two-step immune-fluorescence process: (i) FISH employing double-DIG-labeled LNATM miR-1246 probe, followed by (ii) CD206 immunostaining. Figure 6c shows a clear association between miR-1246 and CD206 macrophages specifically in tumors harboring mutp53 and not WT p53. To exclude false-positive staining from the secondary antibodies, we performed a parallel experiment omitting each time one of the following primary reagents (miR1246 or anti-CD206) (Supplementary Fig. 6c). In addition, we tested the possible role of exosomes as vehicles for miR-1246 in mutp53 tumors. Thus, circulating exosomes were isolated from the plasma of CRC patients. miR-1246 levels were found to be significantly higher in exosomes isolated from plasma samples taken from patients with mutp53 tumor compared with patients whose tumors did not carry mutp53 (Fig. 6d) (*p* < 0.05, Student’s *t* test). As a control, we measured other miRs such as miR-21 and miR-4454, which did not yield any significant difference between the WT +  indels and the mutp53 groups. In this experiment, we also used miR-454 levels as a normalizing factor since it was found to be similar in all samples. Taken together, these results suggested that miR-1246 is transferred from mutp53 tumor cells to the microenvironment including macrophages.

### TGF-β signaling and immunosuppression in mutp53 CRC patients

**Fig. 7:**
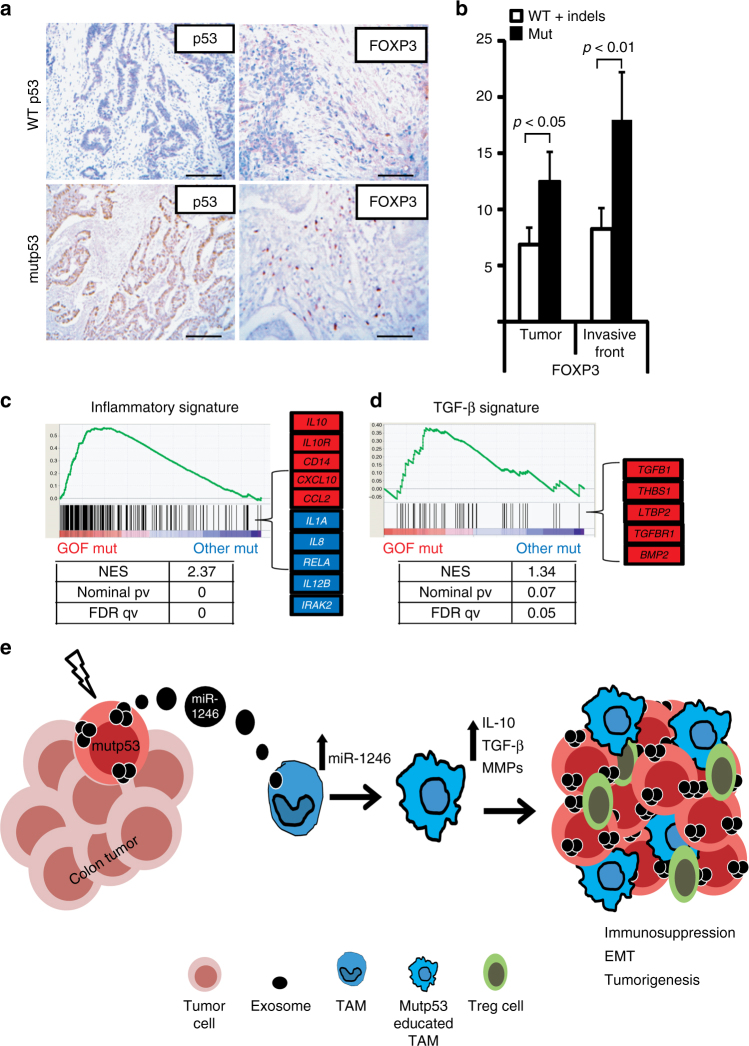
Increased TGF-β signaling and immunosuppression in mutp53 CRC patients. **a** Strong epithelial immunostaining of p53 is correlated with strong non-epithelial immunostaining of FOXP3 in a representative human CRC case carrying mutp53 and compared with a WT p53 case. **b** FOXP3 staining abundance and intensity was calculated for all specimens as described in the Methods section. **c**,**d** A general inflammatory gene set (**c**) and hallmark TGF-β signaling gene set (**d**) produced by a GSEA analysis of mRNA expression levels of the GOF p53 mutants group of CRC tumors compared with the rest of the missense p53 mutants group (other mut). Specific genes significantly upregulated in the GOF group are displayed in red while inflammatory genes downregulated in the GOF group are in blue. NES normalized enrichment score, FDR false discovery rate. GOF mutants refers to the following p53 positions: R245, R248, R175, R273, and R282. **e** The proposed molecular model by which mutp53 facilitates the tumor microenvironment. Colon tumor cell acquires a mutation in TP53 yielding an increased release of exosomes containing miR-1246. Such exosomes are received by neighboring macrophages that undergo a phenotypic shift resulting with enhanced secretion of anti-inflammatoy cytokines (which also recruit immunosuppressive T-regulatory cells) and epithelial−mesenchymal transition (EMT) promoting factors contributing to tumorigenesis and eventually to poor prognosis. Error bars represent standard errors

Since TGF-β, a major inducing source for immunosuppressive T-regulatory (Treg) cells^37^, was shown to be overly secreted by mutp53-reprogrammed TAMs (Supplementary Table 3), we conducted additional IHC for several immune-compartment members. A positive correlation was found between the mutp53-related TAM population and Treg cells depicted by the increased positive staining of FOXP3 in mutp53 tumors compared with the tumors not carrying a missense mutp53 (Fig. 7a, b). The presence of Treg cells in tumors is associated with alternative activation of macrophages^38,39^. Furthermore, in GSEA analysis conducted over the gene expression of the CRC cohort, we could identify several gene sets upregulated in GOF p53 mutants compared with the rest of the p53 mutants. Among the different gene sets (comprehensively presented in Supplementary Table 3) a specific inflammatory signature was found to be significantly altered in the GOF group, including anti-inflammatory genes such as IL-10 and CCL2 while excluding pro-inflammatory genes as *IL1A*, *IL12A*, and *IL8* (Fig. 7c). Specific genes in the TGF-β signaling pathway were also positively correlated with the GOF group including *TGFB1*, *THBS1*, and *LTBP2* (Fig. 7d). Because miR-1246 was able to induce increased secretion of TGF-β from M2 macrophages (Supplementary Fig. 7a), we propose a molecular model (Fig. 7e), where exosomes carrying miR-1246 are being released from mutp53 colon tumor cells. Such exosomes are internalized by neighboring macrophages that, in turn, undergo reprogramming and produce anti-inflammatory and tumor supportive factors including IL-10, TGF-β, and MMPs. These mutp53-reprogrammed macrophages are thus able to induce an anti-inflammatory microenvironment, recruit immunosuppressive Tregs and promote tumor progression.

### SUMOylated hnRNPa2b1 sorts exosomal miR-1246 in mutp53 tumors

To investigate the specific sorting mechanism of miR-1246 into exosomes in mutp53 tumor cells, we performed an RNA:protein pull down assay using biotinylated miR-1246 compared with biotinylated scrambled control. Lysates from WT p53 and mutp53 cells pulled down with biotinylated miR-1246 were analyzed by mass-spectrometry to identify protein associates to miR-1246. This pull down was repeated twice and the averaged results of the top 20 proteins are shown in Supplementary Fig. 7b. Among the proteins pulled down by both WTp53 and mutp53, we identified heterogeneous nuclear ribonucleoproteins A2/B1 (hnRNPa2b1), an RNA binding protein. Interestingly, this protein was reported to recognize a specific motif in miRs and sort them into exosomes but only in its SUMOylated form^40^. Notably, miR-1246 bears such a recognition Exo-motif (Supplementary Fig. 7c). We therefore measured SUMOylation levels of hnRNPa2b1 in HCT116 cells harboring mutp53 and compared with HCT116 harboring WT p53. To do that, we performed co-immunoprecipitation (co-IP) between hnRNPa2b1 and SUMO1. While the general levels of hnRNPa2b1 were higher in the WT p53 cells (IP: hnRNPa2b1), the SUMOylated hnRNPa2b1 was increased by threefold in the mutp53 cells (IP:SUMO1) suggesting a role for this RNA-binding protein in sorting miR-1246 into exosomes (Supplementary Fig. 7d). Finally, to identify potential miR-1246 target genes in macrophages, we transfected M2 macrophages with either miR-1246 mimic, a miR-1246 inhibitor or a mimic control. Then, we performed a gene expression array to explore the potential miR-1246 targets. Following miR-1246 treatment, RNA was extracted and subjected to expression microarray analysis. The heatmap (Supplementary Fig. 7e) depicts the expression patterns of the genes whose abundance was significantly upregulated or downregulated following miR-1246 treatment compared with a control scrambled mimic. As an additional control, this list of genes was also validated in the miR-1246 inhibitor treatment group to be unchanged. The array findings were consistent with previous results as genes such as *CCL2*, *ADAM12*, *MMP2*, and *CCL7* were upregulated. The final list of potential downregulated targets consisted of 36 genes including *IL17A*, *IL7R*, *LEF1*, *S1PR1*, *BCL2*, and *CD96* (full list detailed in Supplementary Table 6).

## Discussion

Modification of the microenvironment allows tumor cells to overcome inhospitable extracellular conditions and progress towards metastasis. To do so, tumor cells utilize cell-to-cell interactions including gap junction channels, paracrine delivery of growth factors and chemokines as well as extracellular vesicles (exosomes and microvesicles)^41^. Recent studies have highlighted the key involvement of exosomes in the facilitation of tumor microenvironment. Exosomes serve as communication vehicles between the tumor cells and their surroundings, favoring secretion of growth factors, cytokines, and angiogenic factors by stromal cells, induction of proliferation of endothelial cells, metastasis, and immune responses^42–45^. Therefore, it is not surprising that mutations in TP53, the most common genetic alteration in human cancer cells are also involved with the process of exosomal transfer.

For over 20 years, the concept of mutant p53 gain-of-function is supported by an increasing amount of published evidence. The ability of specific p53 mutants to promote tumor growth was demonstrated in cells, in animal models and was correlated with clinical prognosis^7–9,46^. Despite ample data accumulated for cell-autonomous mechanisms governed by mutp53, not many direct attempts were conducted to verify any effect on the tumor microenvironment. Several studies, however, do imply that specific mutp53 proteins are capable of a non-cell-autonomous effect^47^. For instance, cancer-associated fibroblasts were shown to secrete higher levels of interferon-β in response to tumor cells harboring mutp53^48^. Recent studies also revealed that molecular events occurring in the WT p53 epithelial cell can also induce a paracrine effect. Notably, WT p53 was suggested to promote an antitumorigenic tissue microenvironment in response to specific signals and mediate an M1 polarization pattern in neighboring macrophages^49,50^. Following similar logic, here we show that mutp53 is also capable of driving a microenvironmental shift that reprograms neighboring macrophages.

The functional link between miR-1246 and cancer is also becoming more evident. Many reports are showing tight association with several types of cancer, including non-small cell lung, colon, breast, cervical and oral squamous cell carcinomas^16,51–53^. miR-1246 was found to promote invasiveness and stemness, to be highly expressed in metastases and proposed to serve as a bio-marker for early detection of certain malignancies. In addition, miR-1246 may target WT p53 in hepatocellular carcinoma where it inhibits cell growth and even reported to be targeted by p53 itself in Down syndrome^54,55^. While these accumulated findings might indicate that miR-1246 is part of a complicated and context-dependent network, the fact that others have found it in cancer-derived exosomes reinforces the argument that miR-1246 is involved in tumor progression and metastasis^29,52^. Still, even though miR-1246 is significantly expressed in mutp53 tumor-derived exosomes, the manner through which miR-1246 is being sorted into the exosomes and how mutp53 is involved in the exosome machinery needs to be further investigated.

In addition, it is worth mentioning that mutp53 GOF is considered mutant-specific. To that end, achievement of successful clonal selection governed by mutp53 might variate depending on the specific mutp53 protein, other genetic events as well as the specific tumor microenvironment conditions. Studying large numbers of human tumors will be instrumental in determining mutant-specific GOF mechanisms.

Understanding how miRs are being sorted into exosomes and deciphering specific recognition motifs are still considered an uncharted territory. RNA binding proteins are likely to play a key role in specifically packaging RNA molecules with a defined recognition. In this study, we followed the discovery of hnRNPa2b1 protein as a key effector in binding to exosome-related motifs on miRs and actively participating in their packaging inside the vesicles^40^. We found hnRNPa2b1 to be pulled down with miR-1246 that contains the specific exo-motif recognized by the protein. The SUMOylated protein is associated with mutp53, leading the way for miR-1246 enrichment in exosomes. This line of experiments needs to be further investigated to reveal the molecular mechanism allowing SUMOylation of hnRNPa2b1 in the cellular context of mutp53.

In summary, colon cancer cells with GOF mutp53 promote the formation of a distinctive population of reprogrammed macrophages by releasing exosomes containing miR-1246. It is yet unclear whether these macrophages are the only neighboring cells affected or, as reported before^56^, other stromal and immune cell types are part of such an interplay. Also, exosomal transfer might not be the only mechanism through which mutp53 tumor cells modulate their microenvironment. Still, these findings highlight the cardinal role played by this microenvironmental cross-talk as oncogenic events occurring in malignant tumor cells exert a paracrine effect which might explain clonal selection advantages for specific GOF p53 mutations eventually resulting with more aggressive carcinomas. Given the fact that therapeutic strategies targeting specific miRs^57^, manipulating exosomes^58^, returning mutp53 to its WT form^59,60^ and shifting M2 macrophages to M1^61^ are all rapidly evolving fields, this study can significantly contribute to such precision cancer therapies.

## Methods

### Cell lines

The following human cells lines were used: HCT116 and DLD-1 (p53 isogenic panel of WT, null and mutp53 received from the lab of Bert Vogelstein in Johns Hopkins University), HT29 (American Type Culture Collection (ATCC)), H358 (ATCC). All cell lines have been authenticated using a short-tandem-repeats^62^ analysis (Frederick National Laboratory for Cancer Research) and verified in STR database (http://strdb.cogcell.org/search_strname.php). HCT116 cells were cultured in Mckoy’s 5A, HT29 cells in DMEM, DLD-1 and H358 were cultured in RPMI 1640. All lines were supplemented with 10% of exosomes-depleted FBS (SBI), the serum for the H358 cells was tetracycline-free (GIBCO). In addition, 100 U ml^−1^ penicillin and 100 μg ml^−1^ streptomycin were added to all media. All cell lines were tested for mycoplasma contamination and kept in a humidifying atmosphere at 5% CO_2_ at 37 °C. HT29 cells were introduced with a stable shRNA transfection with either scrambled non-specific oligos (ShCon) or specific oligos targeting p53 (Shp53). Plasmids were designed with two separated sets of oligos with the following sequences: *ctccactacaactacatgtgta* or cccggcgcacagaggaagagaa and used to knock-down p53 in order to exclude off-target effects. H358 cells were engineered and inserted with inducible transactivation vectors. Full-length p53 cDNA was inserted into pLenti6.3/TO/V5-DEST vector (Invitrogen). Four different mutations (V157F, R175H, R249S, and R273H) were generated by site-directed mutagenesis using the QuikChange II XL site-directed mutagenesis kit (Agilent Technologies). The transactivator was first transduced (pLenti3.3/TR -Invitrogen) and selected (G418). After the stable line was obtained, full-length lentiviral vectors were transduced following a blasticidin selection.

### Monocyte isolation and macrophage culture

Blood was obtained from healthy donors at the NIH blood bank following an informed consent by all subjects and the guideline of the NIH blood bank. PBMCs from buffy coats were isolated using Histopaque-1077 (Sigma-Aldrich) density gradient centrifugation at 400 × *g* for 20 min. Subsequently, PBMCs underwent negative selection for CD14 monocytes using the EasySep™ Human Monocyte Enrichment Kit (StemCell Technologies) according to the manufacturer’s instructions. Selected monocytes were then seeded in six-wells at 5×10^6^ per well in monocytes attachment medium (PromoCell). After 1 h, non-adherent cells were removed by repeated washing and the remaining adherent fraction was cultured in three different manners as follows: (1) In DXF medium (Promocell) for M0 non-activated macrophages. (2) In M1-activation medium (Promocell) for M1 classically activated macrophages. (3) In M2-activation medium (PromoCell) for M2 alternatively activated macrophages. After 6 days additional boost of fresh media was added together with LPS + IFN-γ (50 ng ml^−1^ for M1 macrophages), IL-4 (50 ng ml^−1^ for M2 macrophages) or no additional cytokines (for M0 macrophages). After 3 more days (day 9) media were replaced and cytokines were added as in day 6. All experiments were conducted between days 10 and 12.

### Co-culture of tumor cells and macrophages

Isolated monocytes were seeded in the lower chamber of a six-well transwell apparatus with 0.4-μm pore size (Corning, Lowell, MA, USA) and differentiated to macrophages as described above. After 6 days, tumor cells (HCT116, HT29, H358, or DLD-1) were seeded in the upper inserts and co-cultured for additional 3–5 days. Cell densities were as follows: HCT116—1.5×10^4^ cells/insert, HT29— 5×10^4^ cells/insert, H358—3×10^4^ cells/insert, and DLD-1—3×10^4^ cells/insert.

### Exosome isolation from cells and plasma

Cells were grown in T162 cm^2^ flasks (3×10^6^ per flask) for 5–6 days. Next, the media was collected and centrifuged at 500 × *g* for 5 min, followed by a centrifugation step of 15,000 × *g* for 30 min to discard cellular debris. Subsequently, the media were filtered using a 0.22-μm pore filter (Steriflip, Millipore). The collected media were then ultracentrifuged at 100,000 × *g* for 1.5 h at 4 °C. The exosomes pellet was washed with 24 ml PBS, followed by a second step of ultracentrifugation at 100,000 × *g* for 1.5 h at 4 °C. Afterwards, the supernatant was discarded. Exosomes used for RNA extraction, TEM, and NTA were resuspended in 50 μl PBS. Five microliters of these exosomes sample were used for NanoSight LM10 (NanoSight Ltd) analysis after dilution 1:100 in PBS. Exosomes used for protein extraction were resuspended in 20–30 μl of RIPA lysis buffer. For exosomes isolated from plasma samples taken from human patients—frozen plasma was thawed on ice, 500 μl of cell-free plasma was diluted in 24 ml PBS and filtered through a 0.2-μm pore filter. Afterwards, the samples were treated as mentioned above.

### Nanoparticle tracking analysis

Nanoparticle tracking analysis measurements were performed using a NanoSight NS500 instrument (NanoSight NTA 2.3) following the manufacturer’s instructions. Samples were processed in duplicates and diluted with PBS (1:100) before analysis. NTA post-acquisition settings were optimized and kept constant between samples, three videos 60 s long were recorded per sample and were analyzed to give the mean, mode, and median particles size together with an estimate of the number of particles.

### Electron microscopy

Exosomes were prepared as described previously^63^. Briefly, exosomes were fixed with 2% paraformaldehyde, adsorbed to carbon film grids, fixed with 1% glutaraldehyde, washed with water, and then stained with uranyl-oxalate. The samples were then embedded in a mixture of 9:1 of 2% methylcellulose and 4% uranyl acetate. The embedded samples were then analyzed by electron microscopy. Images were recorded on a Hitachi H7650 TEM with a 2k × 2k AMT CCD camera.

### Optiprep density gradient

Discontinuous iodixanol gradients (6 ml each), containing 40, 20, 10, and 5% solutions of iodixanol were prepared by diluting a stock solution of OptiPrep™ (60%) aqueous iodixanol (Sigma-Aldrich) with 0.25 m sucrose/10 mm Tris, pH 7.5. Each exosomal preparation was individually overlaid on the gradient, and centrifugation performed at 100,000 × *g* for 18 h at 4 °C. Eight individual 3 ml gradient fractions (with increasing density) were collected manually and specified F1 through F8. Fractions were diluted with 20 ml of PBS and centrifuged at 100,000 × *g* for 1.5 h at 4 °C and resuspended either in 50 μl of PBS or 20 μl of RIPA buffer depending on downstream application.

### Western blotting

Cells and exosomes were lysed in RIPA buffer (Cell Signaling). Sample loading was normalized according to BCA protein assay kit (Pierce) and proteins were separated following an electrophoretic gradient across polyacrylamide gels. Wet electrophoretic transfer was used to transfer the proteins in the gel onto 0.45 µm pore-size PVDF membranes (Novex). The protein blot was blocked for 1 h at room temperature with starting block (Thermo) and incubated overnight at 4 °C with the following primary antibodies: anti-Calnexin (Abcam, ab22595), Anti-Alix (Cell Signaling, 3A9), anti-TSG-101 (Abcam, ab83), anti-CD9 (SBI, ExoAb-kit-1). All antibodies were diluted 1:1000. Afterwards, horseradish peroxidase (HRP)-conjugated secondary antibodies (EMD Millipore, 12-348 and 12-349, 1:5000) were incubated for 40 min at room temperature. Washes after antibody incubations were done on an orbital shaker, three times at 10-min intervals, with TBS-Tween20. Blots were developed with the Dura and Pico supersignal chemiluminescent reagents (Thermo). Uncropped versions of all western blot images are presented at the end of the Supplementary Information accompanying this article.

### TP53 sequencing

DNA was extracted from FFPE tissue samples using the QIAmp DNA FFPE Tissue kit (Qiagen) according to the manufacturer’s protocols. Genomic libraries were prepared using the Tagged Amplicon Sequencing methods, previously described^64^. Libraries were quantified with KAPA qPCR library quantification kit, and sequenced on an MiSeq or HiSeq Sequencers (Illumina) using 150 bp or 125 bp paired-end sequencing protocols. Reads were de-multiplexed according to sample-specific barcodes, and aligned to the reference genome (hg19) with BWA (0.7.5a). Mutations were called and quantified with custom calling pipelines as previously described^65.^

### RNA isolation and quantitative PCR

RNA of cells and exosomes was isolated using the miRNeasy Micro Kit (Qiagen) according to the manufacturer’s protocol. RNA was quantified using a Nanodrop ND-1000 (Thermo Fisher Scientific) and was reverse-transcribed using TaqMan MicroRNA Reverse Transcription Kit for miRNA assay (Applied Biosystems, Foster City, California) or the High capacity cDNA reverse transcription kit (Applied Biosystems). Quantitative-PCR was performed using TaqMan assays (Applied Biosystems, Foster City, California) according to the manufacturer’s instructions with the 7500 HT Real-Time RT-PCR System (Applied Biosystems, Foster City). All TaqMan probes were purchased from Applied Biosystems. All microRNAs were normalized to RNU48 using the −ΔCt method, while mRNA expression was normalized to GAPDH. Both GAPDH and RNU48 were tested for changes between treatment groups and were found to be in similar levels. The following Taqman probes were used:

CXCL8 (IL-8) assay ID: Hs00174103m1,

TNF-α assay ID: Hs00174128m1

IL-10 assay ID: Hs00961622m1

CCL2 assay ID: Hs00234140m1

TGFB1 assay ID: Hs00998133m1

VEGFA assay ID: Hs00900055m1

GAPDH assay ID: Hs0278624g1

miR-1246 assay ID:CS6RNBN

miR-4454 assay ID: 461830mat

miR-21 assay ID: Hs04231424s1

RNU48 assay ID:001006RNA was extracted from colorectal cancer tissues using Trizol, according to the manufacturer’s instructions and analyzed on the Affymetrix U133A arrays. Data were imported and RMA normalized using Partek Genomics Suite 6.5. For miR-1246 mimic/inhibitor array: Total RNA was isolated with miRNAeasy micro kit from Qiagen. RNA quality was checked on Agilent Bioanalyzer. All samples used for microarray analysis had high-quality score (RIN > 9). RNA (100  ng) was reverse transcribed and amplified using GeneChip WT plus Reagent kit following the manufacturer’s suggested protocol. Sense strand cDNA was fragmented and labeled using Affymetrix WT terminal labeling kit. Four replicates of each group were hybridized to Affymetrix mouse (human) Gene ST 1.0 GeneChip in Affymetrix hybridization oven at 45 °C, 60 rpm for 16 h. Wash and stain were performed on Affymetrix Fluidics Station 450 and scanned on Affymetrix GeneChip scanner 3000. Data were collected using Affymetrix AGCC software and analyzed using BRB-ArrayTools software.

### Cytokine array

Proteome Profiler Human Cytokine Array Kit, Panel A (R&D) was used according to the manufacturer’s instructions. Media were collected after 3 days of co-culture.

### Nanostring analysis for miRNA expression

Profiling of exosomal miRNA levels was performed using Nanostring technology (Ncounter Human v2 miRNA Expression Assay) for miRNA analysis of 800 human miRNAs. All sample preparation and processing were performed according to the manufacturer’s protocol. Hybridized probes were purified and counted on the nCounter Prep Station and Digital Analyzer (NanoString) following the manufacturer’s instructions. For each assay, a high-density scan (600 fields of view) was performed. Raw data were normalized to the top 100 miRs using nSolver analysis 2.5 software (Nanostring).

### Immunohistochemistry (IHC)

For IHC analysis the following antibodies were employed: anti-p53 (DO7, sc47698, Santa Cruz; 1:100), anti-CD206 (ab64693, Abcam; 1:1500), anti-Cytokeratin 20 (MA5-13263), anti-CD163 (NCL-CD163, Novocastra-Leica; 1:100), and anti-FOXP3 (ab22510, Abcam; 1:200). IHC was performed on paraffin-embedded tissues. Unmasking of the antigen retrieval was performed by heat-mediated antigen retrieval method in 10 mm citric acid (pH 6.0). The UltraVision LP Detection System was employed (#TL-060-HD, Thermo Scientific, Bioanalytica, Greece) according to the manufacturer’s instructions. For color development 3,3′-diaminobenzidine tetrahydrochloride (Sigma) was employed and hematoxylin was used as counterstain. Evaluation of p53 was performed as previously described^12,62^. The evaluation of CD206, CD163, and FOXP3 was performed by counting the number of positive immune cells per high power field (magnification ×400) within tumor nests in the entire cancerous tissue and separately in the invasive front. Kupffer cells in human liver as well as human placenta served both as positive controls for CD163 and CD206 staining. Human tonsil was employed as positive control for FOXP3 staining. Three independent observers carried out slide examination, with minimal inter-observer variability.

### Mass spectrometry

The conditioned media were collected and concentrated using a 3k MWCO concentrator (Amicon). The proteins were then precipitated using acetone. The protein pellets were solubilized in 6 m urea in 100 mm ammonium bicarbonate and in-solution trypsin digested following reduction and iodoacetamide alkylation. The resultant peptides were desalted on a C18 spin column (Pierce) and lyophilized. Dried peptides were solubilized in 2% acetonitrile, 0.5% acetic acid, 97.5% water for analysis on an Orbitrap Fusion (Thermo) mass spectrometer. Proteome Discoverer 1.4 (Thermo) was used to search the data against human proteins from the UniProt database using SequestHT and MSAmanda. The Percolator node was used to score and rank peptide matches using a 1% false discovery rate.

### Flow cytometry

Macrophages were detached using macrophage detachment medium (Promocell). The single-cell suspensions were stained with antibodies specific for CD14 (BD Biosciences, 1:100), CD163 (BD Biosciences, 1:100), and CD206 (BD Biosciences, 1:100). Isotype control for each antibody was used in each experiment as reference. Approximately 1×10^5^ events were collected for each sample on a FACSCalibur (Becton Dickinson), dual laser, flow cytometer using CellQuest Pro Software (BD Biosciences), and analyzed using FlowJo software (Tree Star Inc, CA, USA).

### Phagocytosis assay

After being co-cultured with tumor cells as indicated in the co-culture section, macrophages were harvested using macrophage detachment solution (Promocell) and re-seeded onto an eight-chamber slide (Lab-tek) at a density of 50×10^4^ cells/well. On the following day, medium was removed from the wells and replaced with fresh medium containing Zymosan A BioParticles (Alexa-488, Molecular Probes) at a ratio of 100 particles/cell for 2 h. Macrophages were then washed with cold PBS, fixed in 4% PFA, mounted and analyzed for phagocytized particles using a fluorescence microscope.

### Invasion/migration assays

Impedance measurement was performed with an ACEA xCELLigence^®^ Real-Time Cell Analyzer (RTCA) DP (Roche Diagnostics, Mannheim, Germany). All experiments were performed following the manufacturer’s instructions. In brief, the gold-film electrodes deposited on the upper chamber of the “CIM-plate 16” electrode arrays were coated with matrigel for 2 h for the invasion assay while no matrigel was used for migration monitoring. Cells were seeded at a density of 5000–10,000 cells/well.

### Extra cellular matrix degradation assay

We performed this assay using the QCM™ Gelatin Invadopodia Assay according to the manufacturer’s instructions. Briefly, post co-culture, macrophages were removed using a macrophages detachment solution (Promocell) and seeded onto cy-3 gelatin-coated eight-wells chamber slides. Cells were cultured on the gelatin matrix for 24 h and then stained with FITC-phalloidin and DAPI to visualize cells and gelatin degradation sites. To quantitate the degradation activity, images were taken in a Zeiss LSM 710 confocal microscope (Carl Zeiss) and the Zen light software was used to profile signal ratio between FITC and the Cy-3.

### In situ hybridization

The LNA^TM^ microRNA probe double-DIG labeled (hsa-miR-1246, 610948-360, Exiqon) was employed for visualization of the miR-1246. The LNA^TM^ Scramble-miR (699004-360, Exiqon) and LNA^TM^ U6 snRNA probe (U6, hsa/mmu/rno, 699002-360, Exiqon), double-DIG-labeled probes were used as negative and positive controls respectively. The miRCURY LNA^TM^ microRNA ISH optimization kit FFPE (Exiqon) was employed following the manufacturer’s instructions (instruction manual v3.0). Nuclear Fast Red was used as counterstain. Evaluation was performed by counting the number of positive cancer cells and separately of stromal cells with an intense dark-blue signal per high power field (magnification, ×400).

### Fluorescence in situ hybridization—immunofluorescence

For in situ co-detection we employed sequentially a two-step immune-fluorescence process: (1) FISH utilizing the Locked Nucleic Acid (LNA) technology developed from Exiqon allowing superior hybridization properties for the detection of miR-1246. To further increase detection sensitivity, we used double-DIG-labeled probe (hsa-miR-1246, 610948-360, Exiqon) visualized with Tyramide Signal Amplification (TSA) Plus Fluorescein System (an upgraded version of standard tyramide systems, TSA^TM^ Plus Fluorescein System, NEL741B001KT, Perkin Elmer), (2) IF detecting employing anti-CD206 (ab64693, Abcam) or anti-CD163 (NCL-CD163, Novocastra-Leica). All probes are diluted in 1× microRNA ISH buffer according to the instruction manual v3.0, by Exiqon. For FISH, we employed sheep-anti-DIG-POD (11207733910, Roche) prior incubation with the Tyramide Signal Amplification. For IF we used the following secondary antibodies: Alexa Fluor^®^ goat-anti-rabbit (568) (A-11011, ThermoFisher Scientific, Invitrogen) and Alexa Fluor^®^ goat-anti-mouse (568) (A-11004, ThermoFisher Scientific, Invitrogen) binding to anti-CD206 and anti-CD163 respectively. SlowFade^®^ Gold antifade reagent with DAPI (S36938, Life Technologies) was employed as mounting medium. The positive and negative control of the method was verified employing double-DIG-labeled U6 small nuclear RNA (U6, hsa/mmu/rno, 699002-360, Exiqon) and Scramble-miR (699004-360, Exiqon) probes respectively. To exclude false-positive staining, we performed a parallel experiment omitting each time one of the following primary reagents: miR-1246 or anti-CD206 or anti-CD163.

### In vivo tumor growth

The growth and metastasis of the tumors (HCT116 inserted with mCherry_luciferase vector) were monitored by bioluminescence imaging using the IVIS imaging system. To visualize the bioluminescence signal of tumor cells, mice were injected intraperitoneally with 100 µl of 10 mg ml^−1^ d-luciferin potassium salt (dissolved in PBS) and the bioluminescence images were taken after 10 min. Images were analyzed with the Living-Image software. Regions of interest (ROIs) were indicated by encircling each area of interest including tumors, lungs, and liver, as well as blank background as internal control. Total (sum of signal intensity across entire ROI), average (average of signal intensity at any one point in the ROI), and maximum (greatest signal intensity at any one point within the ROI) radiant efficiencies were recorded for each region of interest.

### Intra-splenic injection

A total of 5×10^5^ HCT116 cells were mixed with 10^5^ M2 macrophages, resuspended in 60 μl PBS and injected at the inferior pole of the spleen using a 29G needle. The bevel of the needle was observed through the splenic capsule to avoid injecting the cells under the spleen. Whitening of the spleen and blood vessels was observed upon injection.

### Statistics

Results are expressed as means ± SEM. Statistical analyses were performed using Graphpad/Prism software. Means of continuous outcome variables were tested with two-tailed Student’s *t* test. Cumulative probabilities of overall survival and relapse-free survival were computed with the Kaplan−Meier method; and log-rank test was used to assess their statistical significance. *P* values less than 0.05 were considered statistically significant.

### Study approval

All protocols used for animal experiments in this study were approved by the NCI-Bethesda ACUC Guidelines/Policies (ACUC No. LHC-010).

### Data availability

Gene expression data have been deposited in the GEOprofiles database under the accession codes GSE44861 and GSE107870. The miR-Chip for exosomes data referenced during the study are available in a public repository from the EVpedia website under the study name “Cooks”. The mass-spectrometry data were deposited in the massIVE database, with accession number MSV000082047. The authors declare that all the other data supporting the findings of this study are available within the article and its Supplementary Information files and from the corresponding author upon reasonable request.

## Electronic supplementary material

Supplementary Information
