# Supplementary material for: Mutant p53 cancers reprogram macrophages to tumor supporting macrophages via exosomal miR-1246
Source: Nat Commun. 2018 Feb 22;9:771. doi: 10.1038/s41467-018-03224-w (PMC5823939; doi:10.1038/s41467-018-03224-w)
Supplement: Supplementary file 1 — Supplementary Information [file 41467_2018_3224_MOESM1_ESM.pdf]

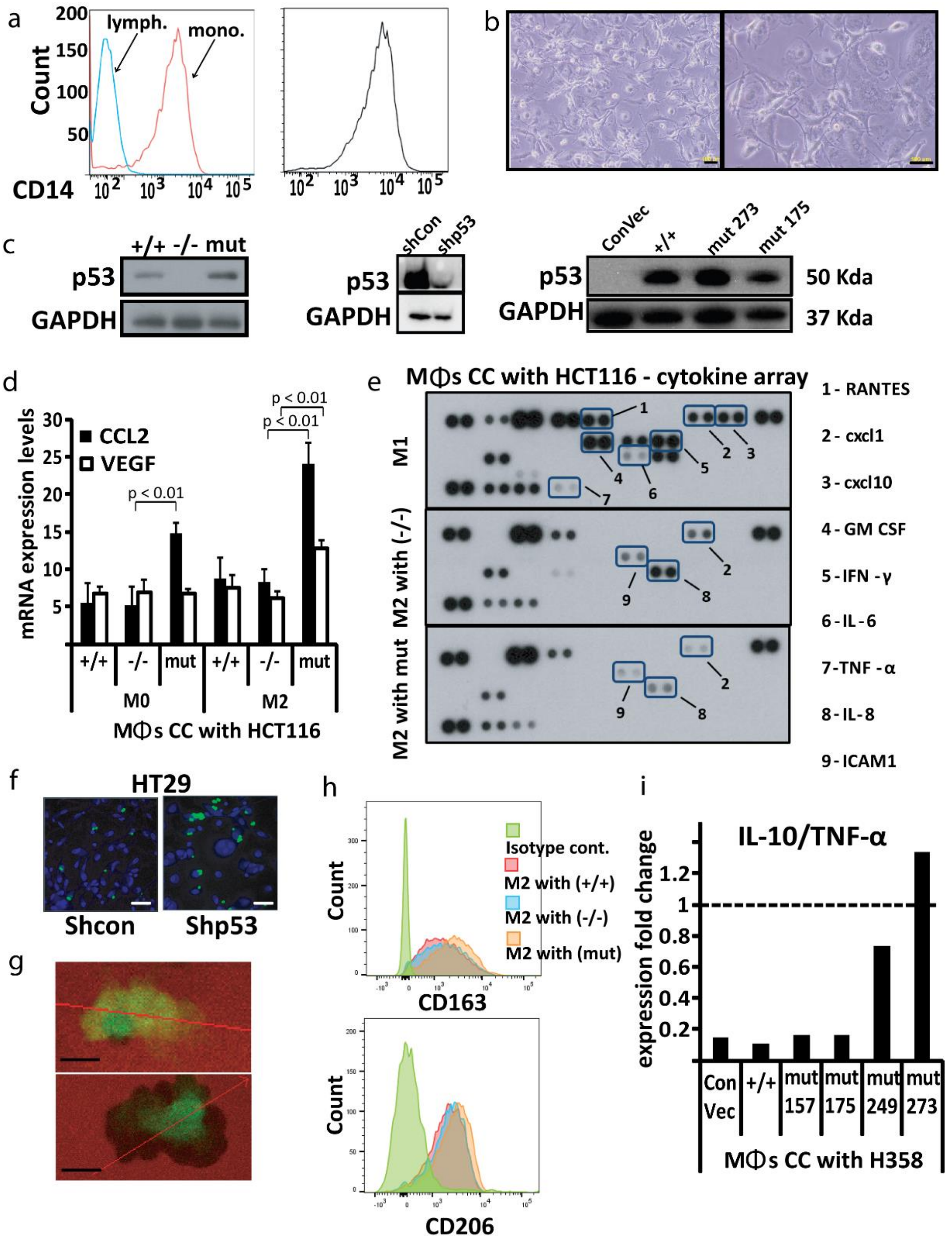

Supplementary Fig. 1– (a) Primary monocytes were purified from blood donations either with a negative selection for CD14 cells (right panel) or without (left panel). The isolated monocytes

were subjected to a flow cytometry analysis using a CD14 antibody. (b) Representative photos of a mature macrophages culture after a negative selection isolation of monocytes and 10 days of culture. Bars= 100 µm. (c) Representative western blot figure presenting protein levels of p53 in HCT116 cells (left panel), HT29 cells (middle panel) and H358 cells (right panel). GAPDH is used as loading control. (d) Matured macrophages were co-cultured with an isogenic set of HCT116 cells differing by their p53 status (+/+ = WT p53, -/- = p53 null, mut = mutp53, p.R248W). RNA was extracted and subjected to qPCR analysis with primers specific to CCL2 and VEGF. (e) Matured M2 macrophages were co-cultured with an isogenic set of HCT116 cells differing by their p53 status (-/- = p53 null, mut = mutp53, p.R248W). After 3 days, Media were collected and used for a proteome cytokine array. M1 macrophages served as a pro-inflammatory control. A numbered index of cytokines is listed to the right of the panel. (f) Representative images of macrophages which were co-cultured with HT29 (with or without mutp53 knock-down, p.R273H) and later incubated with fluorescent zymosan to evaluate phagocytosis capacity. Bars= 50 µm. (g) Representative images of co-cultured macrophages which were harvested and seeded onto a cy-3-gelatin covered glass slide. Bars= 5 µm. (h) Co-cultured macrophages were harvested, stained with fluorescent antibodies against CD163 and CD206 and analyzed by flow cytometry. Relative intensities were compared to isotype controls. (i) Matured macrophages were co-cultured with a doxocycline-induced set of H358 cells differing by their p53 status (Con Vec = p53 null, +/+ = WT p53, mut 157 = V157F p53 mutant, mut 175 = R175H p53 mutant, mut 249 = R249S p53 mutant, mut 273 = R273H p53 mutant). RNA was extracted and subjected to qPCR analysis with primers specific to IL-10 and TNF-α.

Columns depict the ratio between IL-10 expression and TNF-α expression in each sample normalized to GAPDH.

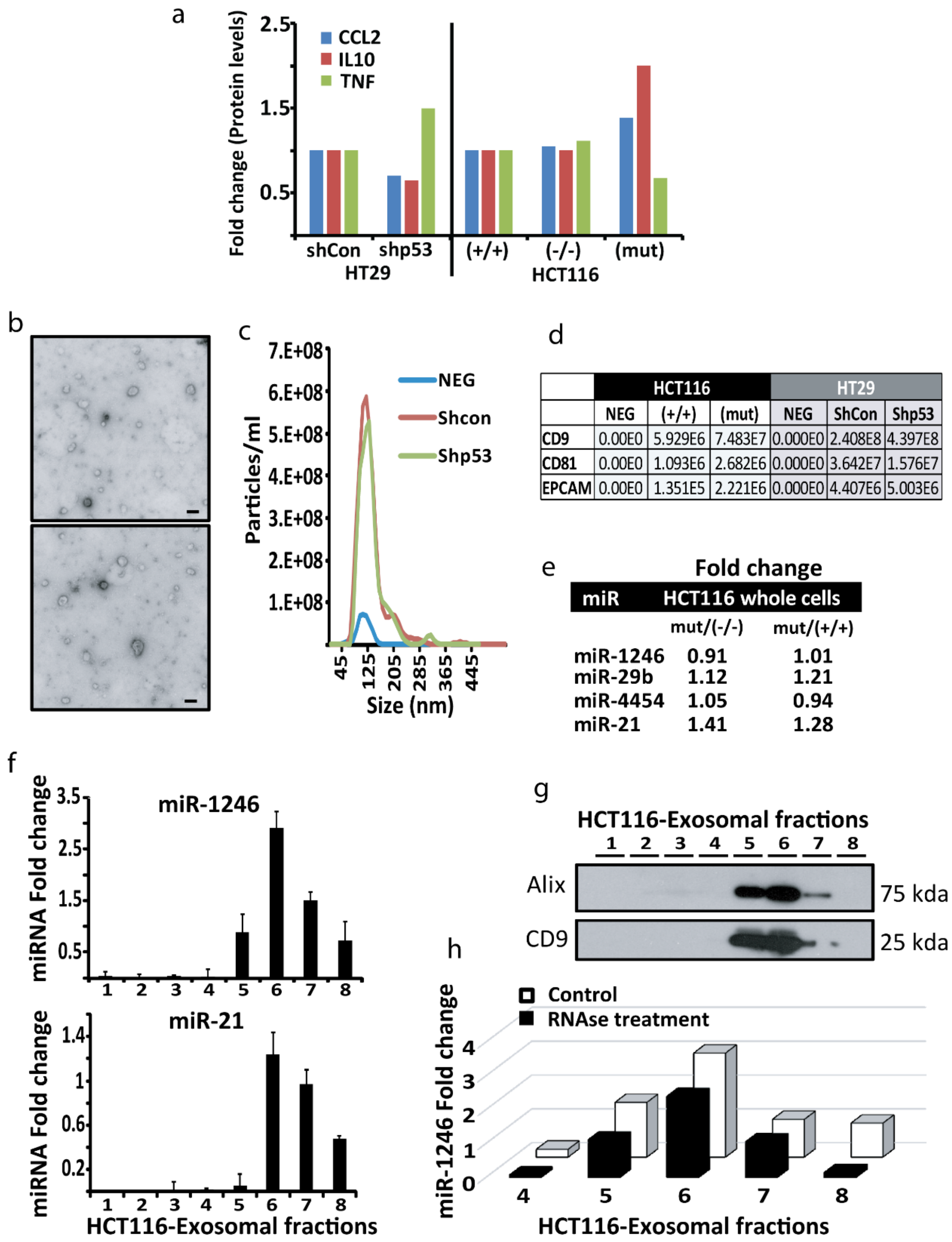

Supplementary Fig. 2- (a) Relative changes in protein secretion levels measured by ELISA assay for CCL2, IL-10 and TNF-α in HCT116 and HT-29 cells differing by p53 status. (b) Representative transmission electron microscopy (TEM) images of paraformaldehyde (PFA)-fixed exosomes on formvar grids at 80,000× magnification. Scale bar = 100 nm. (c) NTA analysis for size distribution and concentration of exosomes isolated from HT29 (ShCon or Shp53) compared with isolations from cell-free medium (NEG). (d) Mass spectrometry analysis of the precursor ion intensities of the three indicated exosomal proteins identified in exosomes isolated from either HCT116 (+/+, mut) or HT29 (ShCon, Shp53) cells. (e) RNA was extracted from HCT116 cells (p53 +/+, -/- or mut) and levels of the miRs indicated in the table were measured using qPCR. The table compares fold changes in the indicated miRs between HCT116 mut and HCT116 +/+ or HCT116 -/-. (f,g) Exosomes from HCT116 cells were isolated using an ultracentrifugation optiprep density gradient separation. Fractions were subjected to qPCR analysis with primers specific to miR-1246 and miR-21 (f) as well as to western blot analysis with the indicated antibodies for exosomal markers (g). (h) Exosomes from HCT116 cells were isolated using an ultracentrifugation optiprep density gradient separation. Before RNA was extracted, exosomes were treated with RNAse A (final concentration 0.5 mg/ml) for 20 minutes at 37°C to degrade unprotected RNA compared with a control treatment with no RNAse A.

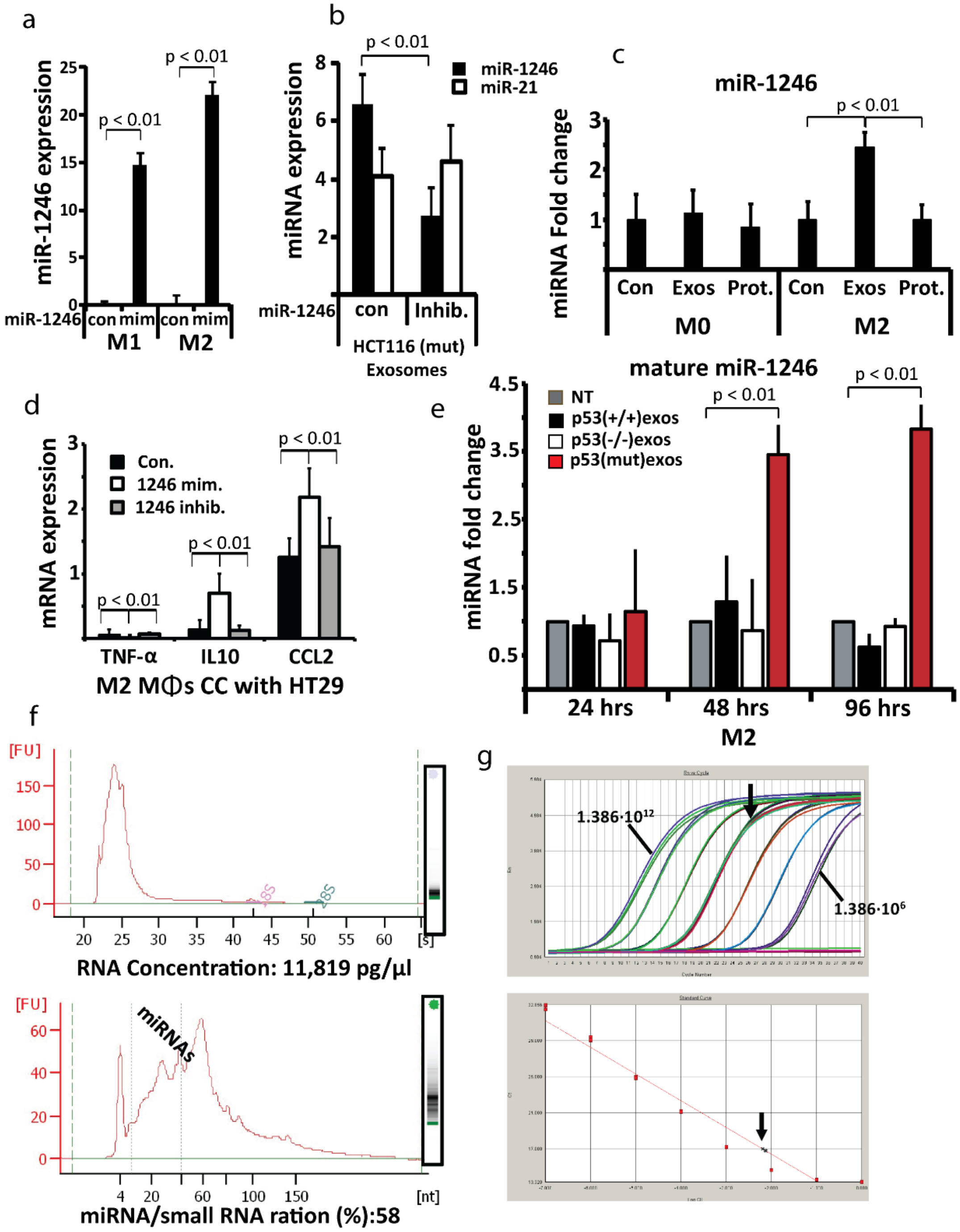

Supplementary Fig. 3 – (a) M1 and M2 Macrophages were transfected with LNA-miR-1246 mimic (mim) and compared to macrophages transfected with an equivalent control vector (con). RNA was extracted from the macrophages and subjected to qPCR analysis with primers

specific to miR-1246 to verify mimic efficiency. (b) HCT116 cells (p.R248W) were transfected with miR-1246 inhibitor (Inhib.) and compared to macrophages transfected with an equivalent control vector (con). Three days post transfection, exosomes were isolated from the transfected cells, exosomal RNA was extracted and miR-1246 and miR-21 levels were measured using qPCR. (c) Exosomes were isolated from HCT116 (mutp53) cells and further separated from free proteins by size-exclusion. M2 macrophages were added either with the exosome fraction (Exos), the protein fraction (Prot.) or PBS as negative control (Con). RNA was extracted after 48 hours and miR-1246 levels were measured. (d) HT29 mutp53 (R273H) cells were transfected with LNA-miR-1246 mimic (mim) or miR-1246 inhibitor (inhib.) and compared to an equivalent control vector (con). Forty-eight hours later, cells were co-cultured with M2 macrophages for an additional 3 days after which RNA was extracted from the macrophages and subjected to qPCR analysis with primers specific to TNF-α, CCL2 and IL-10. (e) M2 Macrophages were grown in the presence of 10 μg exosomes isolated from HCT116 cells differing by their p53 status for different time periods. RNA was extracted from the macrophages and subjected to qPCR analysis with primers specific to miR-1246 normalized to RNU48. (f) Total RNA bio-analyser assay (top panel) and small RNA bio-analyser assay (bottom panel) of secreted exosomes from HCT116 (mutp53). (g) Standard curve obtained for absolute miR-1246 quantification. MiRNA -1246 mimic was diluted to a concentration range of $1.386 \cdot 10^{12}$–$1.386 \cdot 10^{6}$ copies per reaction. Each point was plotted is an average of triplicate fluorescence values for each standard concentration measured. MiR-1246 levels from 10μg (protein) of HCT116 derived exosomes (mutp53) are marked with an arrow.

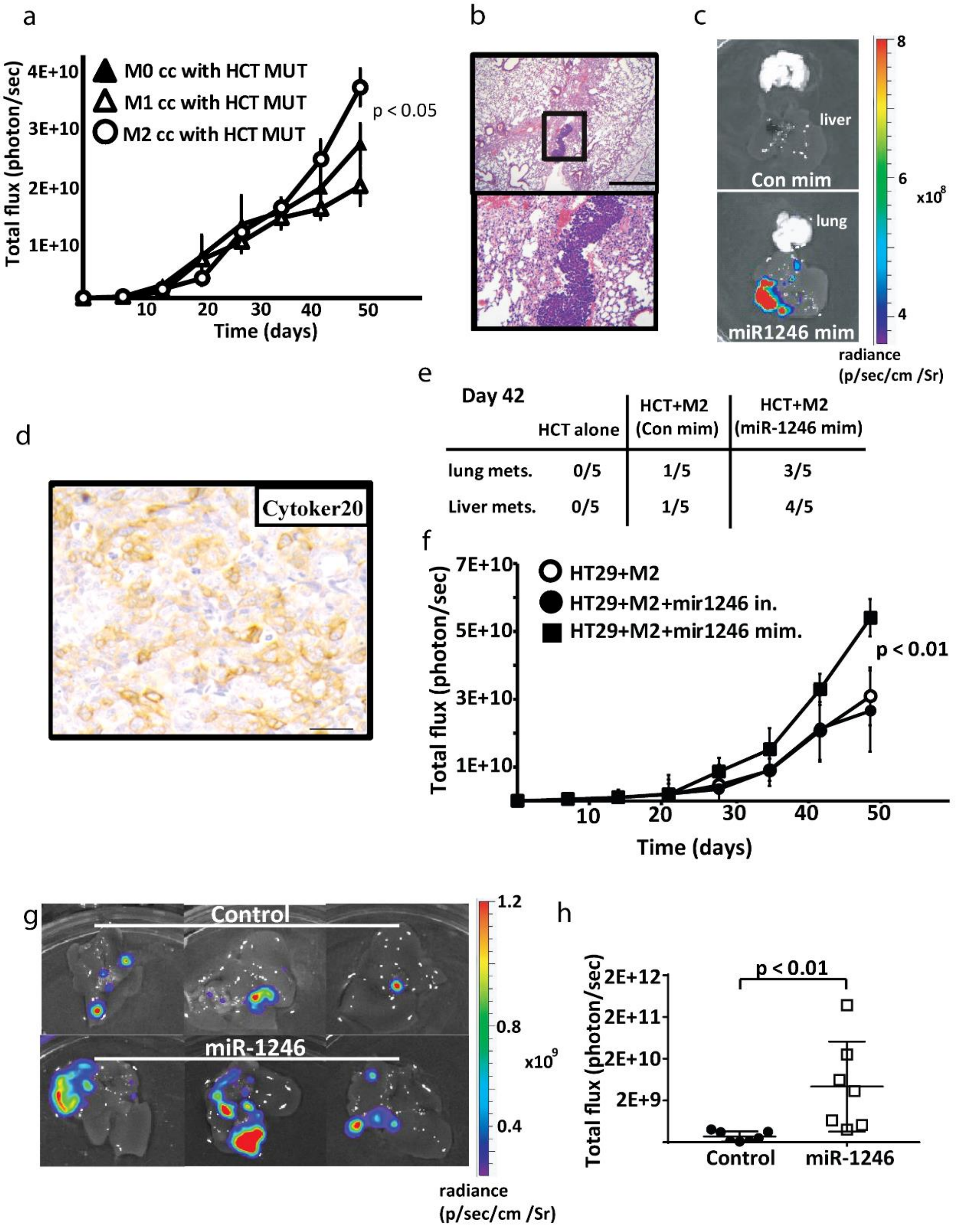

Supplementary Fig. 4 – (a) M2 Macrophages were co-cultured with mutp53 HCT116 cells for 6 days. Subsequently, 10^5 reprogrammed macrophages were mixed with $5x10^5$ fresh HCT116 WT cells (carrying a luciferase vector) and co-injected subcutaneously into NOD-SCID mice. Each

group consisted of 5 animals. Tumor development was monitored weekly. P value is valid only for day 49 (student's t-test) between the M2 and M0 groups. (b) Representative histopathological validation of metastases-bearing lungs. Bars= 100 µm (c) M2 Macrophages were co were transfected with LNA-miR-1246 mimic (mim) and compared to M2 macrophages transfected with an equivalent control vector (con). Subsequently, 10^5 macrophages were mixed with $5x10^5$ HCT116 cells (carrying a luciferase vector) and co-injected subcutaneously into NOD-SCID mice. On day 42, mice were sacrificed and their liver and lungs were monitored for metastatic foci as presented in (c). The table in (e) shows the number of organs observed with metastases, also compared with a group of mice injected with HCT116 cells alone. (d) Cytokeratin 20 immunostaining demonstrating positive immunostaining in mutp53 HCT116 as xenograft tumors into NOD-SCID mice. Tumors were removed and fixed 27 days post injection. Bars=50µm. (f) M2 macrophages were transfected with LNA-miR-1246 mimic or miR-1246 inhibitor and compared to an equivalent control vector. Three days later, the transfected macrophages were mixed with $5·10^5$ luciferase expressing HT29 cells and co-injected subcutaneously to the back of NOD-SCID mice (n=7 for all groups). Mice were monitored weekly for tumor growth using an IVIS imager and luminescent fluxes were quantified. (g,h) M2 macrophages were transfected with LNA-miR-1246 mimic and compared to an equivalent control vector. Three days later, the transfected macrophages were mixed with 10^5 luciferase expressing HCT116 cells and used for an intra-splenic injection to seed metastatic foci directly into the liver (n=7 for both groups). Mice were monitored weekly for metastatic development using an IVIS imager (g), then sacrificed at day 28 and luminescent fluxes were quantified (h).

a

Case ID	p53 status
10186	p.R248Q
11181	p.G244C
11303	p.D281E
11692	p.G245A
11916	p.R175H
11924	p.V173M
12124	p.R175H
12139	p.R273C
12146	p.E349K
12294	p.G245S
12298	p.R273H
12385	p.P278T
12447	p.R282W
12663	p.R248Q
12724	p.R248Q
12742	p.C176F
12861	p.G244C
13201	p.K132R
13273	p.F270L
13285	p.P278L
13687	p.R248W
13822	p.A84A
14022	p.Y205F
14445	p.P278L
14467	p.G245D
14554	p.G245R
14559	p.E346K
15527	p.H193Y
15556	p.E258K
10212	WT
11642	Del
12076	WT
12375	WT
12462	p.R213X
12464	WT
12531	WT
12792	Ins
12829	WT
12874	WT
12879	WT
13799	WT
14273	Del

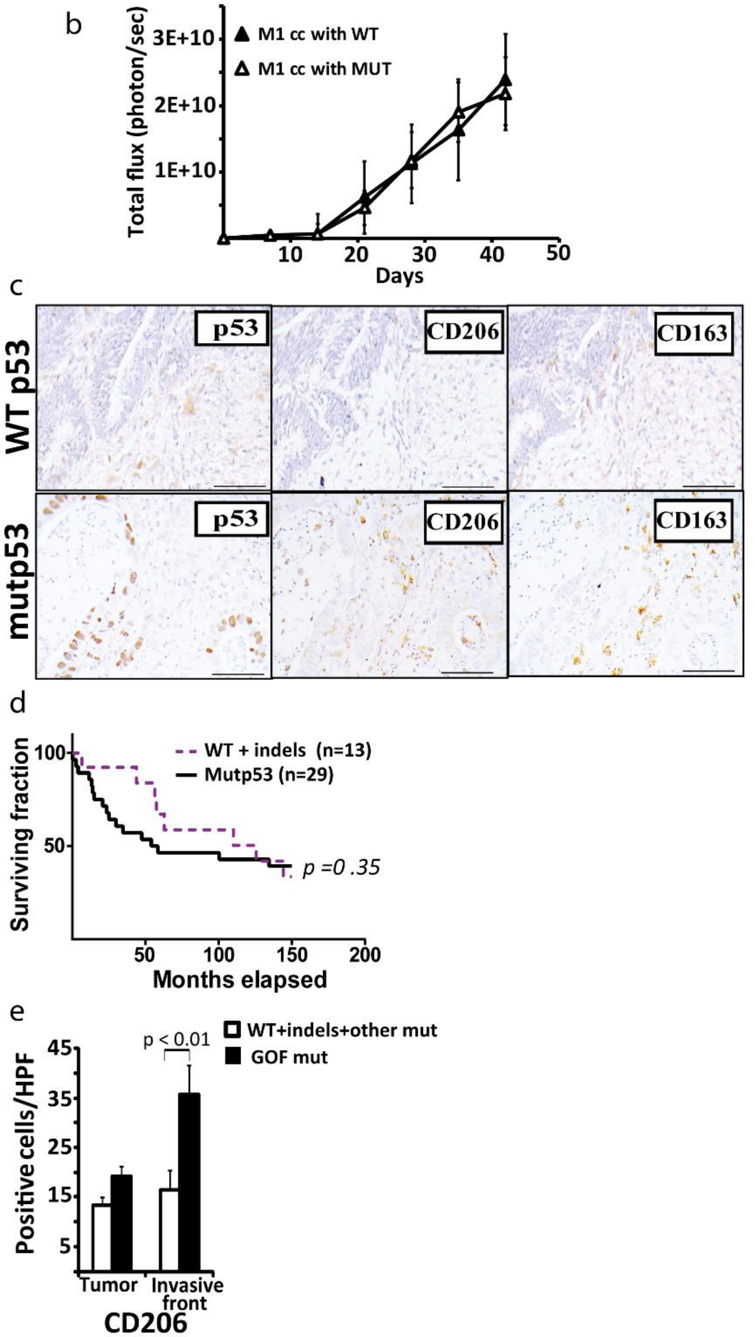

Supplementary Fig. 5 -(a) TP53 sequencing of individual human CRC cases. (b) M1 Macrophages were co-cultured with either mutp53 or WT p53 HCT116 cells for 6 days. Subsequently, 10^5 reprogrammed macrophages were mixed with $5x10^5$ fresh HCT116 WT cells (carrying a luciferase vector) and co-injected subcutaneously into NOD-SCID mice. Each group consisted of

7 animals. Tumor development was monitored weekly. (c) Strong epithelial immunostaining of p53 is correlated with strong non-epithelial immunostaining of CD163 and CD206 in a representative human CRC case carrying mutp53 and compared with a WT p53 case. Bars represent 100µm. (d) Survival curve of CRC patients divided by TP53 status comparing WT+indels with all missesnse p53 mutations (Mutp53). (e) Staining abundance and intensity for CD206 in the tumors or in the invasive front regions. Cases were grouped by p53 status, with non-GOF missense p53 mutations combined with the “WT+ indels” group and compared with the GOF mutants (GOF mut).

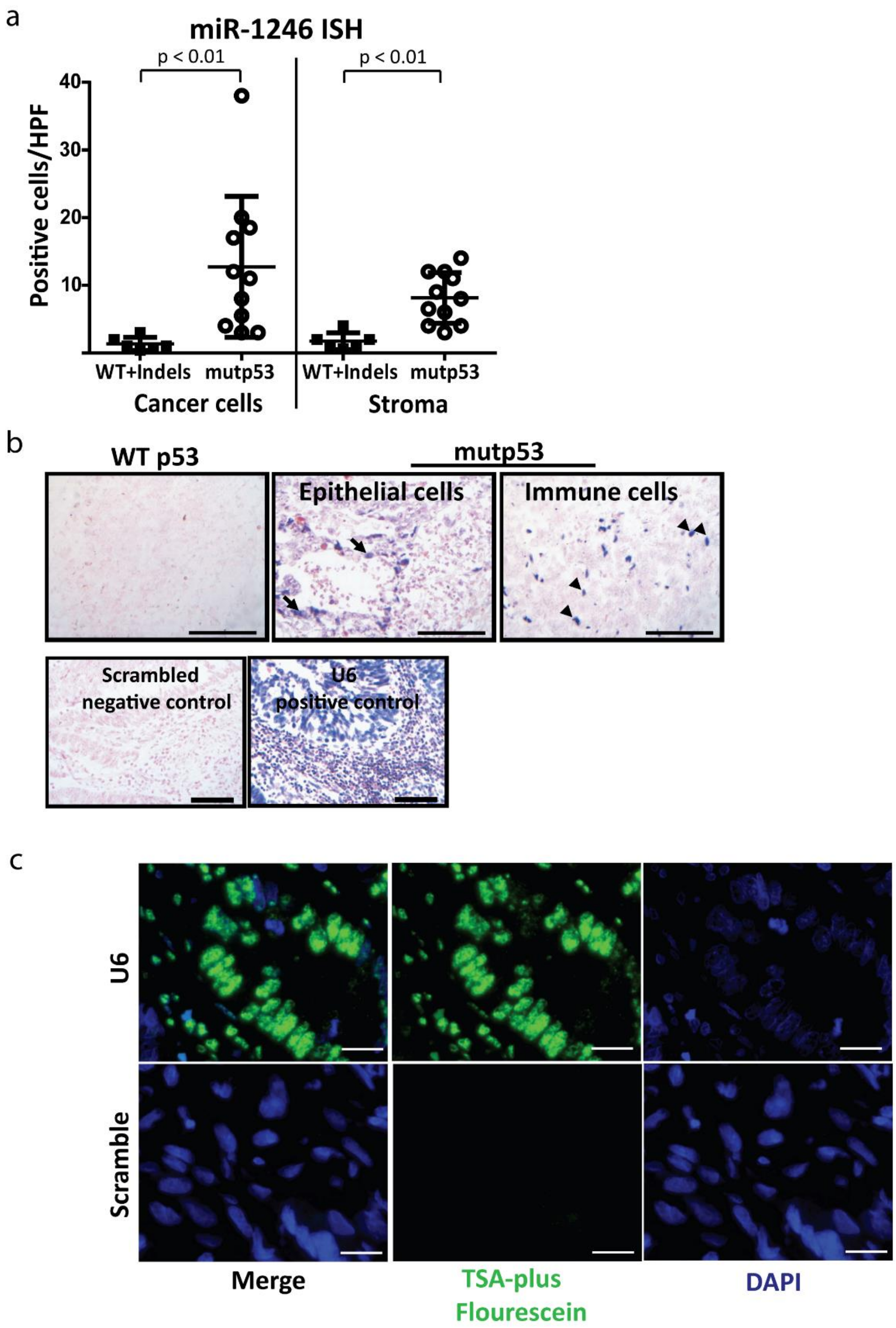

Supplementary Fig. 6 - (a) In-situ hybridization using miR-1246 specific probe was employed on 6 WT p53 tumors and 11 mutp53 tumors. Positive signal was evaluated for epithelial and

stromal compartments of each tumor. (b) In-situ hybridization (ISH) of miR-1246 in CRC tumor harboring missense mutp53 compared with WT p53 tumors. Arrows present epithelial cancer cells, arrowheads present stromal cells. Bars= 100 µm. A scrambled-miR probe was used as negative control while U6 snRNA probe was used as positive control. All probes underwent similar hybridization procedure. Bars= 100 µm. (c) A scrambled-miR probe was used as negative control while U6 snRNA probe was used as positive control. All probes underwent similar hybridization procedure. Bars= 25 µm.

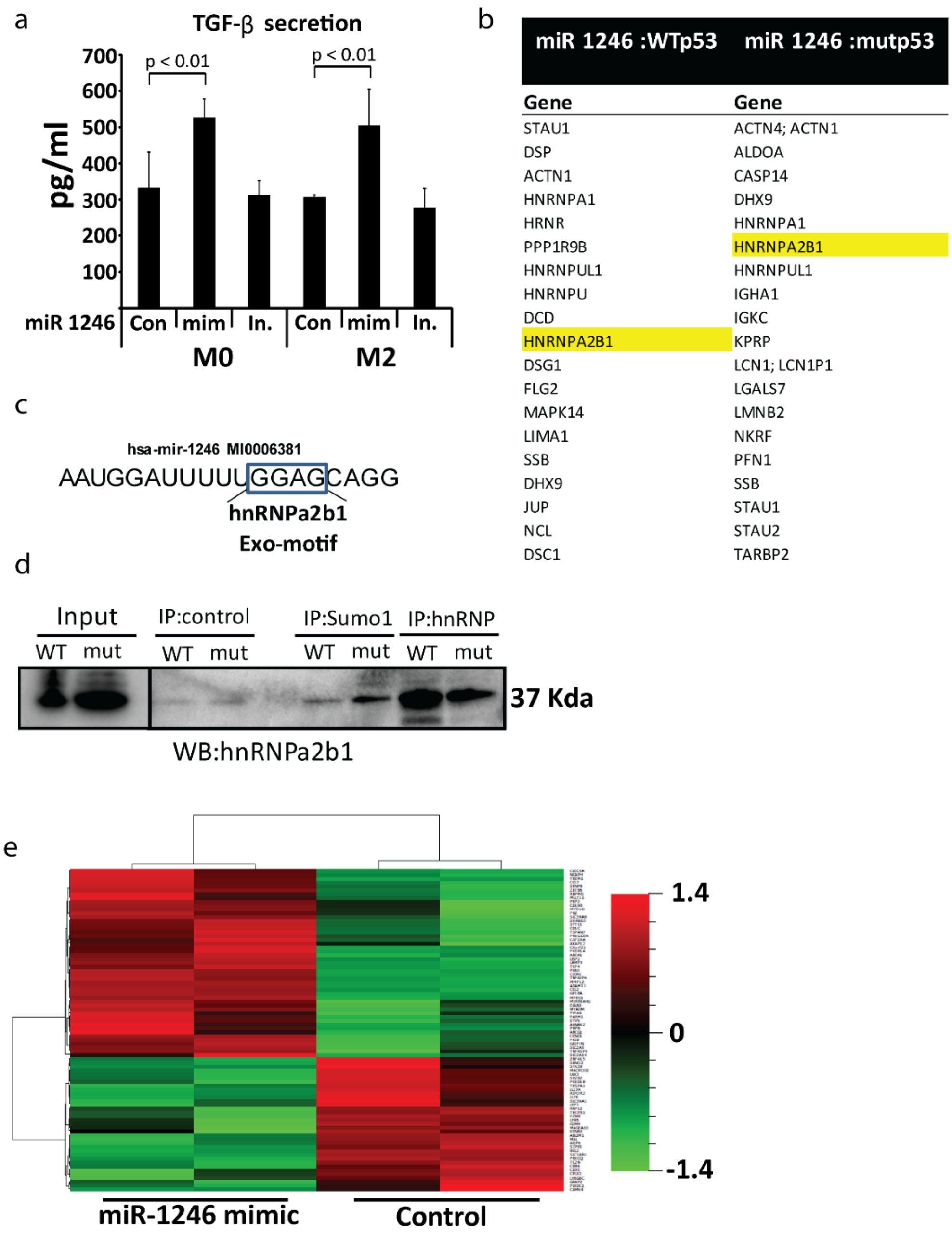

Supplementary Fig. 7 - (a) M0 and M2 macrophages were transfected with LNA-miR-1246 mimic (mim) or miR-1246 inhibitor (in.) and compared to an equivalent control vector (con). Three days later, media were collected and subjected to a specific TGF-β ELISA measurement per the manufacturer instructions. (b) Biotinylated miR-1246 was pulled down with protein lysates from HCT116 cells either harboring WT p53 or mutp53. Proteins pulled down with miR-

1246 were analyzed using mass-spectrometry. Top 20 represented proteins are displayed in the table. (c) MiR-1246 sequence containing the EXO-motif recognized by hnRNPa2b1. (d) Co-immunoprecipitation (co-IP) assay with lysates from HCT116 cells (either WT p53 or mutp53). Lysates were precipitated with either SUMO1, hnRNPa2b1 or a control antibody (IgG) and subsequently blotted with hnRNPa2b1 antibody. Samples from the total lysates served as loading input (Input). (e) M2 macrophages were transfected with LNA-miR-1246 mimic or miR-1246 inhibitor and compared to an equivalent control vector. Three days later, RNA was extracted and subjected to a gene expression array. A heat-map of upregulated and downregulated genes following the treatment of miR-1246 mimic is presented. A complementary list of genes is shown in table S6.

Gene	Fold Changes (M2/M1)
TIMP3	43.0092
CD36	18.6145
MMP7	17.3353
APOE	15.8141
CCL22	13.4366
MSR1	11.871
IL17RB	7.40679
LGALSL	6.80989
IL10	4.14402
CCL18	4.09964
TGFBI	3.41223
CCL2	2.87243
STAT1	-4.04543
IRF1	-4.34677
CXCL1	-6.09048
TLR3	-8.35405
IL8	-3.14482
CXCL5	-14.3717
CCR7	-19.8363
CCL5	-26.0804
IL1B	-32.9034
IL6	-93.3568

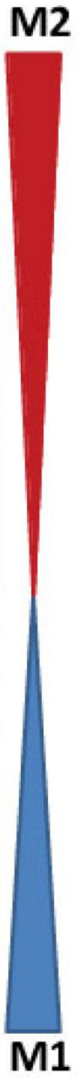

Supplementary table 1- Comparison of Gene expression array values between primary M1 and M2 macrophages. M2 associated genes in red, M1 associated genes in blue.

M2 CC with HCT116			
	no	p53(+/+)	p53(mut)
APOE	0.00E+00	0.00E+00	1.14E+06
ART4	0.00E+00	0.00E+00	1.02E+06
CASC1	0.00E+00	0.00E+00	1.60E+06
CCL18	3.90E+07	1.40E+08	1.10E+06
EXOC5	0.00E+00	0.00E+00	2.37E+08
IGHA1	3.35E+06	8.20E+06	0.00E+00
LRG1	3.86E+06	8.20E+06	0.00E+00
MMP9	1.36E+06	3.98E+06	2.05E+07
SPARC	5.60E+06	6.91E+06	0.00E+00
TGFBI	1.84E+06	2.96E+06	2.63E+07
TMEM198	3.03E+06	4.00E+06	1.55E+07
VNN1	0.00E+00	0.00E+00	7.04E+06
PCDHGA7	0.00E+00	0.00E+00	2.00E+05

Supplementary table 2 - Mass spectrometry analysis of the proteins secreted into media collected from M2 macrophages after being co-cultured with HCT116 cells harboring mutp53 (p53 (mut)), HCT116 cells harboring WT p53 (p53 (+/+)) or not co-cultured (no). The table is comprised of selected proteins that were determined to be significantly different in abundance in the mutp53 sample based on precursor ion signal intensity.

Gene set	Size	ES	NES	Nom. pv	FDR-qv
ALLOGRAFT REJECTION	200	0.632	2.676	0	0
INTERFERON GAMMA RESPONSE	198	0.604	2.554	0	0
EPITHELIAL MESENCHYMAL TRANSITION	200	0.593	2.537	0	0
INFLAMMATORY RESPONSE	200	0.575	2.468	0	0
IL6 JAK STAT3 SIGNALING	87	0.613	2.327	0	0
TNFA SIGNALING VIA NFKB	200	0.487	2.082	0	0
IL2 STAT5 SIGNALING	200	0.436	1.884	0	0
KRAS SIGNALING	200	0.411	1.754	0	0.001
INTERLEUKIN RECEPTOR ACTIVITY	19	0.726	2.034	0	0.002
UV RESPONSE	144	0.418	1.715	0	0.002
PANCREAS BETA CELLS	40	0.515	1.701	0.004	0.002
MYOGENESIS	200	0.395	1.685	0	0.002
COMPLEMENT	199	0.389	1.674	0	0.002
INTERFERON ALPHA RESPONSE	97	0.426	1.651	0.002	0.003
CHEMOKINE RECEPTOR BINDING	42	0.553	1.861	0	0.008
EXTRACELLULAR MATRIX REMODELING	27	0.608	1.838	0.006	0.009
TGF BETA SIGNALING	54	0.383	1.342	0.077	0.055
HYPOXIA	200	0.31	1.326	0.015	0.059
APICAL JUNCTION	200	0.312	1.323	0.019	0.058
COAGULATION	138	0.298	1.186	0.128	0.182
IL10 SIGNALING	17	0.473	1.232	0.185	0.185

Supplementary table 3 – A list of gene-set signatures upregulated in CRC tumors harboring GOF p53 mutations (positions R245, R248, R175, R273, R282) compared with tumors harboring other p53 missense mutants. Significance is depicted by normalized enrichment score, false discovery rate (FDR), and p,q values.

microRNA ID	p-value	Fold Change (Mutp53/WT)
hsa_miR_1246	0.045	2.29
hsa_miR_135b	0.040	2.04
hsa_miR_429	0.147	1.55
hsa_miR_183	0.084	1.54
hsa_miR_96	0.121	1.47
hsa_miR_144	0.199	1.41
hsa_miR_494	0.185	1.39
hsa_miR_200b	0.245	1.37
hsa_miR_32	0.078	1.36
hsa_miR_19a	0.154	1.34
hsa_miR_194	0.287	1.34
hsa_miR_200a	0.215	1.31
hsa_miR_95	0.222	1.30
hsa_miR_92a	0.147	1.30
hsa_miR_221	0.109	1.29
hsa_miR_708	0.157	1.29
hsa_miR_141	0.326	1.27
hsa_miR_21	0.081	1.26
hsa_miR_335	0.223	1.24
hsa_miR_532_3p	0.249	1.24
hsa_miR_532_5p	0.131	1.23
hsa_miR_99b	0.143	1.23
hsa_miR_98	0.095	1.23
hsa_miR_107	0.276	1.23
hsa_miR_362_3p	0.101	1.23
hsa_miR_125a_5p	0.116	1.21
hsa_miR_452	0.213	1.20
hsa_miR_378	0.280	-1.20
hsa_miR_561	0.024	-1.20
hsa_miR_10b	0.321	-1.20
hsa_miR_497	0.410	-1.21

hsa_miR_155	0.200	-1.21
hsa_miR_1297	0.237	-1.22
hsa_miR_1260	0.181	-1.22
hsa_miR_320a	0.091	-1.23
hsa_miR_627	0.095	-1.23
hsa_miR_495	0.164	-1.23
hsa_miR_361_3p	0.050	-1.24
hsa_miR_600	0.074	-1.25
hsa_miR_196a	0.247	-1.26
hsa_miR_215	0.306	-1.27
hsa_let_7b	0.005	-1.27
hsa_let_7c	0.194	-1.29
hsa_miR_490_5p	0.368	-1.29
hsa_miR_143	0.407	-1.30
hsa_miR_10a	0.184	-1.30
hsa_miR_214	0.216	-1.32
hsa_miR_1	0.503	-1.32
hsa_miR_744	0.034	-1.33
hsa_miR_29c	0.032	-1.35
hsa_miR_363	0.320	-1.35
hsa_miR_383	0.112	-1.35
hsa_miR_9	0.134	-1.36
hsa_miR_1274b	0.089	-1.37
hsa_miR_145	0.291	-1.38
hsa_miR_720	0.081	-1.39
hsa_miR_582_5p	0.028	-1.41
hsa_miR_99a	0.193	-1.41

Supplementary table 4 - Profiling of miRNA expression of RNA extracted from 55 CRC tumors subjected to groups of WT (n=27) or mutp53 (n=28). After background subtraction, samples with good overall signal were normalized to the geometric mean of the top 75 miRNAs within each sample. Fold changes of over 1.2 (+/-) are presented in the table.

microRNA	HCT116 (+/+)	HCT116 (-/-)	HCT116 MUT
**hsa-miR-1246**	82.75	68.46	533.13
**hsa-miR-4454**	90.41	77.29	185.95
**hsa-miR-23a-3p**	40.41	86.52	159.47
**hsa-miR-100-5p**	34.03	66.25	147.11
**hsa-miR-125b-5p**	19.85	50.66	131.22
**hsa-miR-191-5p**	21.98	82.62	115.34
**hsa-let-7a-5p**	17.73	50.66	102.98
**hsa-miR-25-3p**	34.03	42.09	91.8
**hsa-miR-29b-3p**	14.18	31.18	88.86
**hsa-let-7b-5p**	21.98	34.29	88.27
**hsa-miR-222-3p**	29.78	56.12	80.03
**hsa-miR-630**	34.74	70.93	77.09
**hsa-miR-200c-3p**	23.4	32.74	64.73
**hsa-miR-302d-3p**	57.43	75.6	57.08
**hsa-miR-21-5p**	7.12	18.71	52.96
**hsa-miR-93-5p**	13.47	26.5	47.08
**hsa-let-7g-5p**	21.27	24.16	40.6
**hsa-miR-720**	9.93	16.37	37.66
**hsa-miR-200b-3p**	9.93	11.69	27.66
**hsa-miR-612**	26.23	21.04	27.07
**hsa-miR-320e**	28.36	28.06	25.89
**hsa-miR-150-5p**	12.76	10.91	24.71
**hsa-miR-148b-3p**	17.73	17.93	24.71
**hsa-miR-135b-5p**	9.22	6.24	24.13

Supplementary table 5 – A full microRNA-chip for miRs expression in exosomes derived from HCT116 cells harboring no p53 (-/-), WT p53 (+/+) or mutp53 (MUT). Values were normalized using the Nsolver analysis software and presented as absolute units of relative expression. The top 25 mutp53 associated miRs are presented out of the full list.

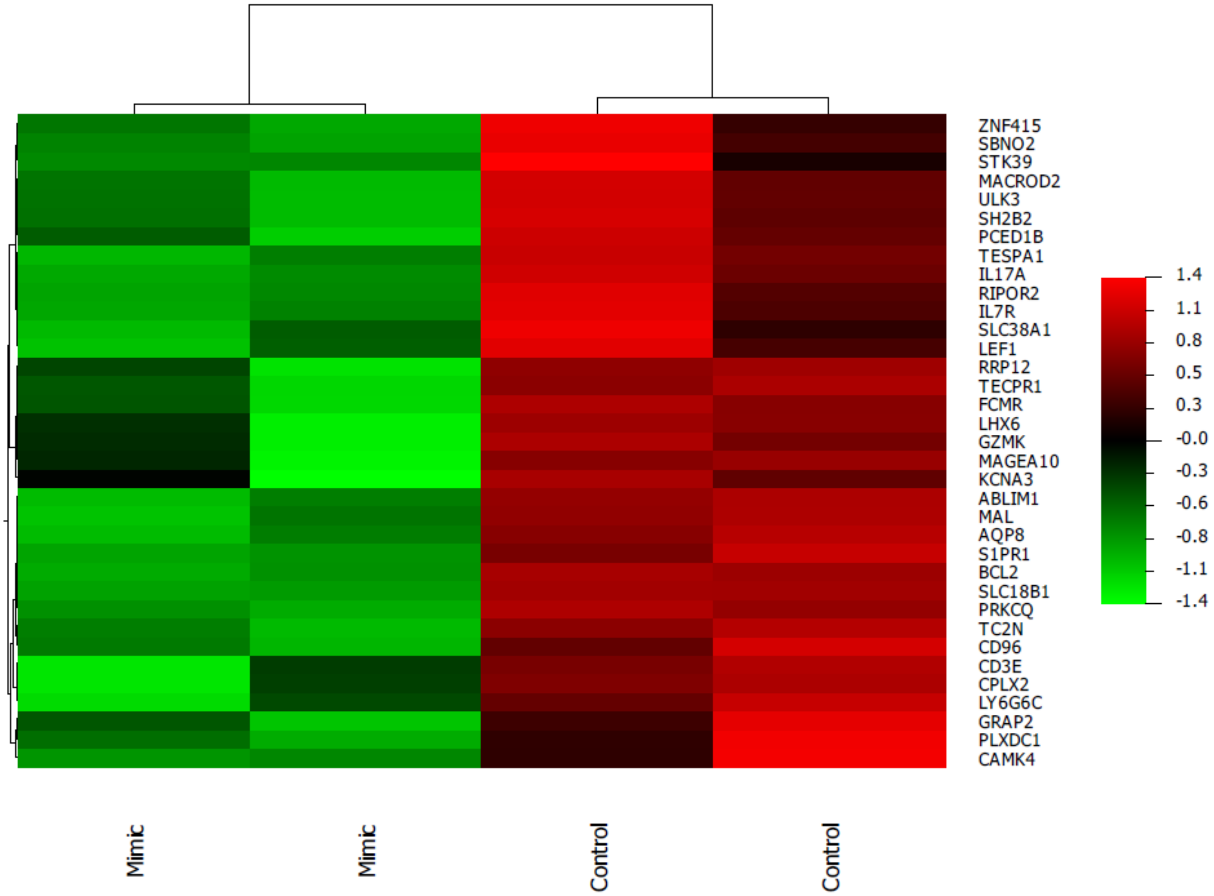

Supplementary table 6 - A list of downregulated genes comprised of a gene expression array analysis performed on M2 macrophages transfected with miR-1246 mimic (mimic). Genes were considered downregulated after comparison with M2 macrophages transfected with a scrambled control (Control).

Uncropped western blot images:

Figure 2a:

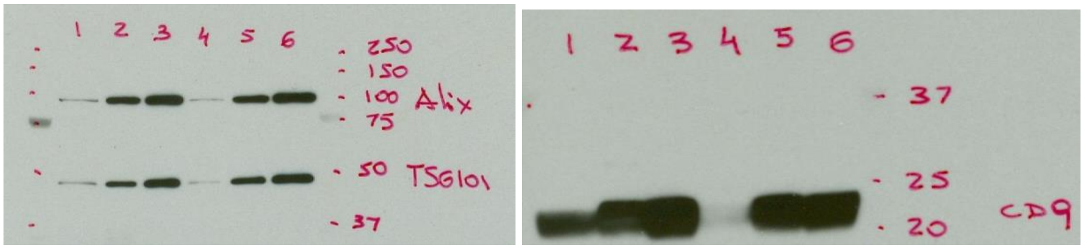

Supp. Figure 1:

HCT116

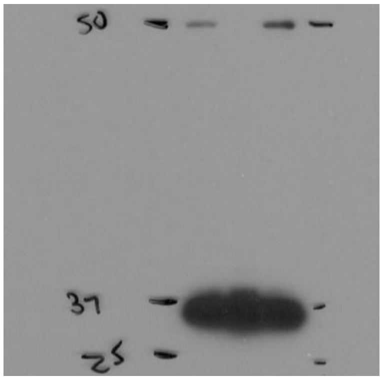

HT29

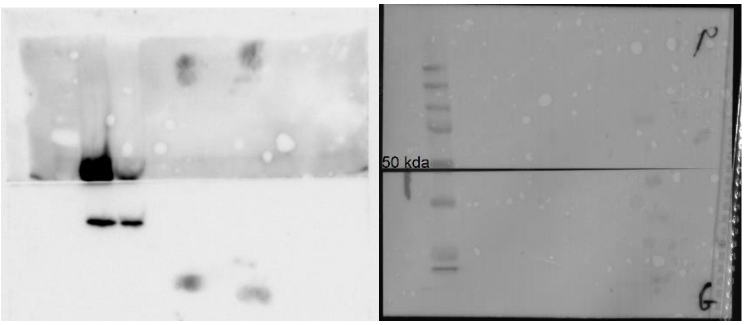

H358

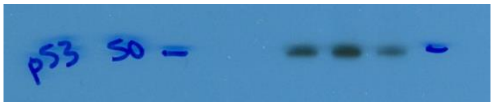

Supp. Figure 2g:

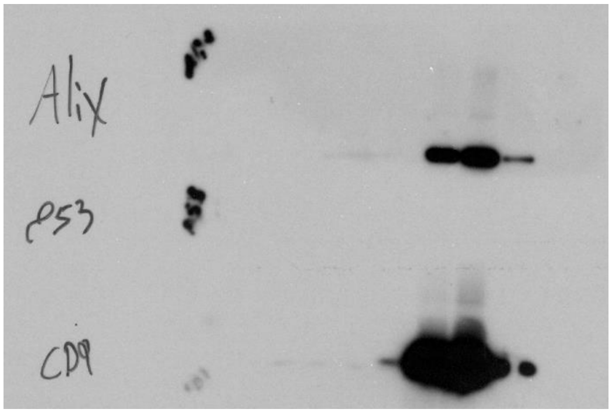

Supp. Figure 7d:

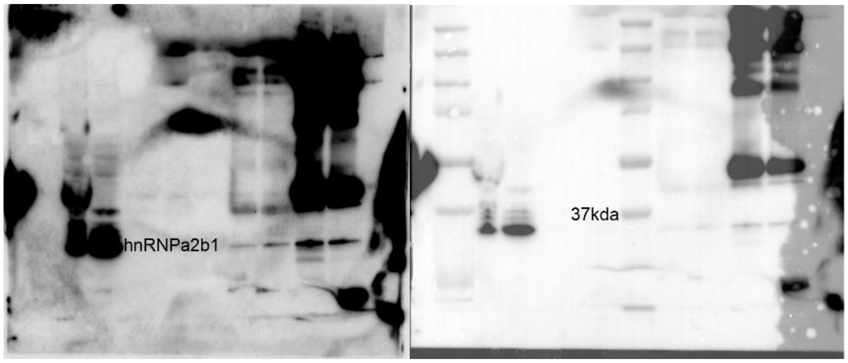
hnRNPa2b1
37kda
